# Effects of Ultra-Processed Meals with Differing Energy Density on Postprandial Glyco-Insulinemic and Appetite-Related Hormonal Responses: A Randomized Crossover Trial

**DOI:** 10.3390/nu18152534

**Published:** 2026-08-04

**Authors:** Isabelle Rodrigues de Souza Gama, Déborah Tenório da Costa Paula, Delis Barbosa Soares, Micnéias Róberth Pereira, Ludmila de Melo Barros, Guilherme César Oliveira de Carvalho, Vanessa Amorim Peixoto, Débora Cavalcante Ferro, Marianna Victoria Cerqueira Rocha, Cellyne Veronica da Silva, Priscila Adelina Alves Viana, Rodrigo Tenório Lins Carnaúba, Thayná Xavier Rocha de Oliveira, José Israel Rodrigues Júnior, Mateus de Lima Macena, André Eduardo da Silva-Júnior, Fabiana Andréa Moura, Nassib Bezerra Bueno

**Affiliations:** 1 Laboratório de Nutrição e Metabolismo (LANUM), Faculdade de Nutrição (FANUT), Universidade Federal de Alagoas, Maceió 57072-900, AL, Brazil; isabelle.gama@fanut.ufal.br (I.R.d.S.G.); deborah.paula@fanut.ufal.br (D.T.d.C.P.); delis.soares@fanut.ufal.br (D.B.S.); micneias.pereira@fanut.ufal.br (M.R.P.); ludmila.barros@fanut.ufal.br (L.d.M.B.); guilherme.carvalho@fanut.ufal.br (G.C.O.d.C.); vanessa.peixoto@fanut.ufal.br (V.A.P.); debora.ferro@fanut.ufal.br (D.C.F.); marianna.rocha@eenf.ufal.br (M.V.C.R.); cellyne.silva@fanut.ufal.br (C.V.d.S.); priscila.viana@fanut.ufal.br (P.A.A.V.); rodrigo.carnauba@fanut.ufal.br (R.T.L.C.); thayna.oliveira@fanut.ufal.br (T.X.R.d.O.); mateus.macena@fanut.ufal.br (M.d.L.M.); andre.eduardo@unifesp.br (A.E.d.S.-J.); 2 Postgraduate Program in Nutrition, Faculdade de Nutrição, Universidade Federal de Alagoas, Maceió 57072-900, AL, Brazil; jose.rodrigues@fanut.ufal.br (J.I.R.J.); fabiana.moura@fanut.ufal.br (F.A.M.); 3 Postgraduate Program in Nutrition, Escola Paulista de Medicina, Universidade Federal de São Paulo, São Paulo 04023-062, SP, Brazil

**Keywords:** insulin clearance, ultraprocessed foods, appetite regulation, hormones

## Abstract

**Background/Objectives**: To evaluate the metabolic and hormonal responses after meals rich in and free of ultra-processed foods (UPF), with high or low energy density (ED), in adults without obesity (body mass index [BMI] between 18.5 and 29.9 kg/m^2^, body fat percentage ≤ 30% for men and ≤42% for women). **Methods**: Randomized, crossover-controlled feeding trial. Participants were assigned to four conditions (UPF+/ED+, UPF+/ED−, UPF−/ED+, and UPF−/ED−), each receiving two standardized test meals (breakfast and lunch), matched for energy, macronutrients, fiber, saturated fat, free sugar, and sodium. Blood samples were collected after 12 h fasting, and 240 min after each meal completion for quantification of glucose, insulin, triglycerides, ghrelin, and leptin. **Results**: Nineteen participants (9 females; 22 y mean age; 23.5 kg/m^2^ mean BMI) were included. The UPF condition resulted in a statistically significant increase in postprandial circulating insulin concentrations throughout the day (*p* = 0.016), without interactions with meal (*p* = 0.09). No main effects of ED or UPF × ED interactions were observed for any outcome. **Conclusions**: In this exploratory secondary analysis of a randomized crossover trial, acute UPF consumption resulted in higher postprandial circulating insulin concentrations, independently of ED. These findings require confirmation in adequately powered studies. Brazilian Registry of Clinical Trials (ReBEC), registration number RBR-10gfr3fb.

## 1. Introduction

The contemporary environment and dietary factors modulate biological and hormonal signals, thereby favoring the development of metabolic disorders in humans [1,2,3]. In this context, ultra-processed foods (UPFs) have received increasing attention, as they generally present high energy density (ED), high palatability, and high levels of sugar, fat, and sodium [4]. These characteristics influence food intake by hyperactivating the brain reward system and eliciting hedonic neural responses [5,6]. Previous evidence indicates that meals composed of UPFs are consumed at a faster eating rate than those composed of non-UPFs, resulting in energy overconsumption and body weight gain [7,8,9]. It is suggested that this consumption pattern is related to their composition and sensory characteristics, since UPFs tend to be consumed more rapidly, which may delay physiological satiety signals and impair appetite regulation [10,11].

Beyond their effects on food intake and satiety, UPFs may also influence acute metabolic responses. Evidence from controlled feeding trials suggests that diets rich in UPFs can alter markers of glyco-insulinemic homeostasis, although findings remain inconsistent. In the inpatient crossover trial conducted by Hall et al. [8], fasting insulin, HOMA-IR, and HOMA-β were consistently higher following the UPF diet than following the unprocessed diet, although the differences did not reach conventional statistical significance. More recently, Çelik and Ulug [12] reported a significantly greater postprandial insulin total area under the curve after a UPF breakfast, while no differences were observed for glucose or appetite-related peptides. In contrast, Hamano et al. [9] observed unfavorable changes in lipid and hepatic biomarkers after a UPF dietary period, but not in markers of glycemic homeostasis. Finally, Galdino-Silva et al. [7] observed no overall differences in acute glycemic or hormonal responses after consumption of a UPF meal, although exploratory analyses suggested a sex-dependent effect on postprandial leptin concentrations, reinforcing the heterogeneity of physiological responses to UPF exposure. Collectively, these findings suggest that UPF consumption may adversely affect metabolic regulation; however, the specific mechanisms involved remain unclear.

It is noteworthy that, in interpreting these findings, ED emerges as a critical variable because it may influence both satiety-related mechanisms and postprandial metabolic responses [13]. It is suggested that the non-beverage ED may affect satiety signaling by altering gastric emptying rate, as the regulation of food intake is primarily based on the physical volume of food rather than solely on its energy content [14]. Thus, ED has been identified as a predictive indicator of increased energy intake and, consequently, could be an important driver or confounder of the metabolic impacts of UPF intake [14,15]. Nevertheless, despite the growing number of studies investigating the effects of UPFs on food intake, experimental evidence evaluating the specific role of meal ED in both high and low UPF content remains limited. Notably, except for Galdino-Silva et al. [7], the available controlled feeding trials have reported substantial differences in non-beverage energy density (ED) between UPF and non-UPF conditions. In Hall et al. [8], although overall meal ED was matched when beverages were included, the non-beverage ED of the UPF diet was considerably higher than that of the unprocessed diet (2.15 vs. 1.15 kcal/g). Similarly, Hamano et al. [9] reported a non-beverage ED of 2.0 kcal/g during the UPF period and 1.1 kcal/g during the non-UPF period, while Çelik and Ulug [12] reported non-beverage ED values of approximately 3.0 kcal/g and 1.6 kcal/g in the UPF and non-UPF groups, respectively. These differences suggest that non-beverage ED may have contributed, at least in part, to the metabolic and behavioral outcomes observed in previous studies, making it difficult to disentangle the independent effects of food processing from those of energy density.

In addition, few studies have explored how the degree of food processing and ED interact to influence metabolic changes during the postprandial period, while matching meals for macronutrients, fiber, sodium, free sugar, saturated fat, and, especially, non-beverage ED. Therefore, the present study aimed to evaluate metabolic and hormonal changes after meals rich in or free of UPFs, with high or low ED, in adults without obesity, in a randomized, crossover clinical trial. We hypothesized that high ED in a UPF meal would present synergistic effects in poorer post-prandial metabolic outcomes.

## 2. Methods

### 2.1. Ethical Aspects

This study is part of a larger project registered in the Brazilian Registry of Clinical Trials (ReBEC) (available at https://ensaiosclinicos.gov.br/ (accessed on 28 April 2025)) under protocol number RBR-10gfr3fb. The project is entitled “Metabolic effects of diets free of or rich in ultra-processed foods and with high or low energy density in healthy adults: a randomized crossover clinical trial,” whose primary objective is to evaluate ad libitum energy intake. The paper reporting the primary outcome (ad libitum energy intake at dinner) is currently being considered for publication elsewhere. The present study reports the prespecified secondary glyco-insulinemic and appetite-related hormonal outcomes collected as part of the same trial protocol.

The study protocol was approved by the Research Ethics Committee of the Federal University of Alagoas (UFAL) (CAAE: 85014024.5.0000.5013). In addition, the present study is reported in accordance with the Consolidated Standards of Reporting Trials (CONSORT) guidelines [16] (Supplementary Table S1). All participants signed the Informed Consent Form (ICF) before inclusion in the study.

### 2.2. Study Design

This is an analysis of prespecified secondary outcomes from a randomized, 2 × 2 crossover clinical trial with controlled feeding. The study was designed to investigate the independent and combined effects of food processing level (ultra-processed and non-ultra-processed foods) and energy density (high and low) on postprandial metabolic responses. Each intervention group lasted 1 day, with a washout period of at least 3 days. Due to the nature of the investigation, blinding regarding the intervention was not possible. However, participants were not aware of the primary outcome and were informed that the study aimed to investigate the “metabolic effects” of meal consumption.

### 2.3. Participants

Data collection was conducted at the Nutrition and Metabolism Laboratory (LANUM) of the Faculty of Nutrition (FANUT) at the Federal University of Alagoas (UFAL) from May to October 2025. A non-probabilistic convenience sampling method was used. Participants were recruited through campus advertising, via the Instagram pages of FANUT/UFAL and LANUM, and on the UFAL website.

Participants were eligible for inclusion if they were men or women aged 19 and 30 years, with a BMI between 18.5 and 29.9 kg/m^2^, body fat percentage ≤ 30% for men and ≤42% for women as determined by bioelectrical impedance analysis [17], who had maintained stable body weight for at least one month; women with regular menstrual cycles; and who were willing to comply with the study schedule.

Individuals were not included if they were under chronic use of medications (such as antidiabetic drugs, antihypertensives, antidepressants, antiretrovirals, immunosuppressants, or any medication that could interfere with hunger regulation and/or body weight maintenance), presented alterations in fasting blood glucose, blood pressure, or lipid profile, had any diagnosed gastrointestinal disease, had conditions that made anthropometric assessment or measurement of energy expenditure components impossible, were unable to consume the provided diet, or had food intolerances, allergies, or dietary restrictions, as well as smokers, alcohol users, pregnant women, lactating women, or menopausal women.

Participants were excluded if they consumed less than 95% of the meals provided during the intervention, ingested any foods other than those supplied by the research group, or failed to follow the study protocol.

Participants were randomly allocated using the sample() function in R (v. 4.3.1, R Foundation for Statistical Computing, Vienna, Austria). Randomization was completely unconstrained and fully random, without enforcing balanced sequence designs. Nineteen independent random sequences were generated, each consisting of a permutation of 1 to 4 without replacement, corresponding to the four intervention conditions. Sequence assignment followed the order of participant inclusion, and each sequence corresponded to the order of exposure to the different intervention conditions throughout the study (1–4). The sequence was kept by a researcher who had no contact with the participants, ensuring allocation concealment. We did not assess period or carryover effects.

### 2.4. Test-Condition

**Figure 1:**
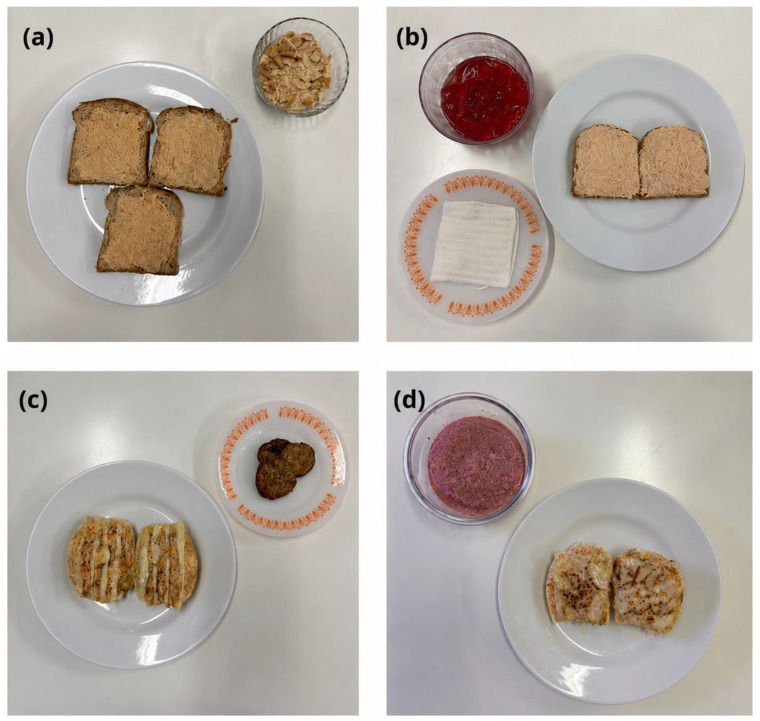
Breakfast from the 2000 kcal intervention group. (**a**) UPF+/ED+: bread with chicken pâté and banana with cookie flour. (**b**) UPF+/ED−: bread with chicken pâté and sweet tapioca with banana and strawberry gelatin. (**c**) UPF−/ED+: bread with chicken pâté and homemade banana cookie. (**d**) UPF−/ED−: bread with chicken pâté and strawberry ice cream.

**Figure 2:**
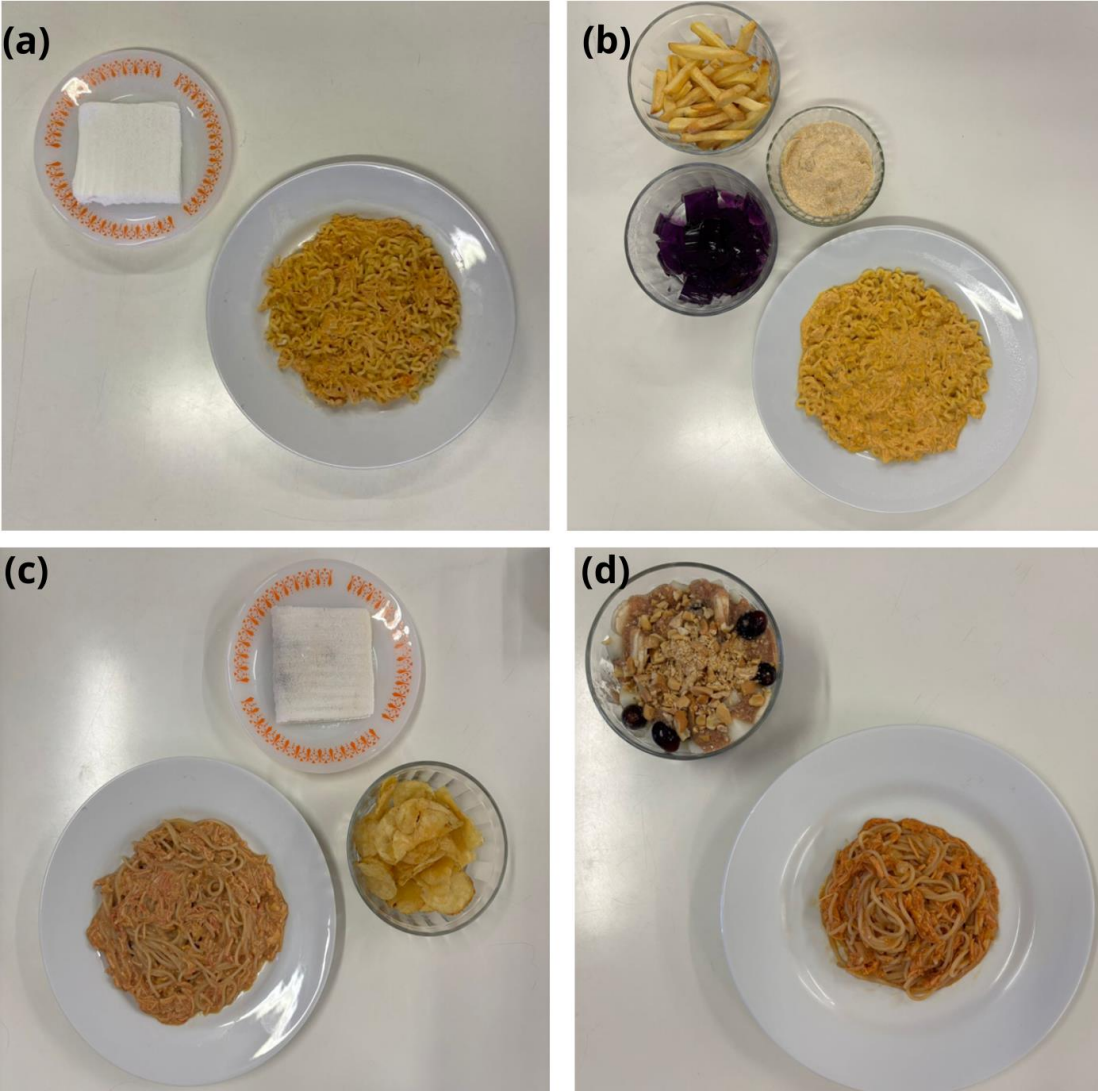
Lunch from the 2000 kcal intervention group. (**a**) UPF+/ED+: creamy chicken spaghetti and sweet tapioca with crunchy chocolate. (**b**) UPF+/ED−: creamy chicken spaghetti, French fries, grape gelatin, and banana with condensed milk. (**c**) UPF−/ED+: pasta with chicken, potato chips, and tapioca with cheese and guava paste. (**d**) UPF−/ED−: pasta with chicken and fruit salad.

Four intervention arms were investigated, consisting of two standardized meals (breakfast and lunch): (1) UPF+/ED+: meals with ≥80% of energy derived from UPFs and high ED; (2) UPF+/ED−: meals with ≥80% of energy derived from UPFs and low ED; (3) UPF−/ED+: meals free of UPFs and high ED); and (4) UPF−/ED−: meals free of UPFs and low ED. Energy density ranged from 1.03–1.20 kcal/g in ED- and 2.0–2.23 kcal/g in ED+ meals (Figure 1 and Figure 2, Table 1).

The energy content of the meals corresponded to 20–25% (breakfast) and 40–45% (lunch) of each participant’s total energy intake (TEI). TEI was determined individually using indirect calorimetry (MetaCheck, Korr, Salt Lake City, UT, USA), which provided resting energy expenditure (REE), and a triaxial accelerometer (ActiGraph wGT3X-BT, ActiGraph LLC, Pensacola, FL, USA), which estimated the metabolic equivalent of task (MET) of the individuals. Using participants’ REE and MET values, the factorial method was applied to determine each individual’s TEI [18]. The energy intake provided was adjusted to achieve neutral energy balance.

All meals were matched for energy, protein, carbohydrates, lipids, fiber, saturated fat, free sugar, and sodium, with a maximum variation of 10% of total energy intake (TEI) for each component between groups (Table 1). The meals were designed using the WebDiet software (WebDiet Health Manager, version 4.0, Rio de Janeiro, Brazil). For the nutritional composition of the foods used in the meals, a hierarchy of preference for nutritional information was applied: (1) manufacturer-provided nutritional information; (2) Brazilian Food Composition Table (TBCA); (3) Brazilian Institute of Geography and Statistics (IBGE) Food Consumption Nutritional Composition Table; (4) United States Department of Agriculture (USDA) FoodData Central database. The macronutrient distribution of the meals followed the Dietary Reference Intakes for the 19–30 years age group, while saturated fat (10% of TEI), fiber (25 g/day), and free sugar (10% of TEI) values followed World Health Organization recommendations [18,19,20,21,22,23]. For sodium, the average habitual intake of the Brazilian population (~3736 mg/day) was used as a reference for an energy requirement of 2000 kcal [24]. For the other energy ranges, a sodium density of 1868 mg/1000 kcal was applied. In addition, standardized preparation sheets were developed for all meals, with energy ranges of 1400–3200 kcal, with 2000 kcal as the reference value.

### 2.5. Study Procedures

**Figure 3:**
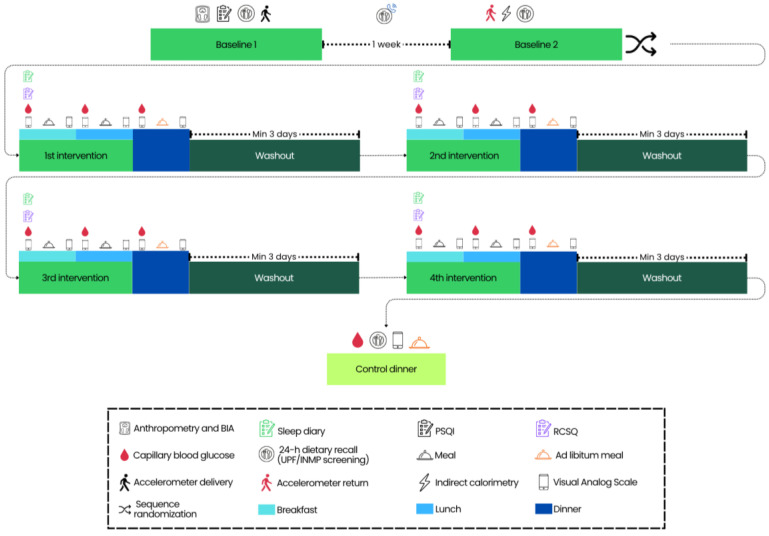
Flowchart of the study design of the parent project.

Figure 3 illustrates the flow diagram of the overall study design, encompassing participant data collection from baseline through the final intervention period. Individuals who expressed interest in participating in the study completed a simple remote questionnaire developed by the researchers using Google Forms (Google LLC, Mountain View, CA, USA). Those who met the inclusion criteria were invited to attend an in-person screening visit to formalize participation in the study. During enrollment, the full flowchart of the larger project and the composition of the meals to be provided were presented.

During baseline assessments, participants were instructed to fast for 12 h. Accordingly, anthropometric measurements (weight, height, waist circumference, and BMI) and body composition assessments using bioelectrical impedance analysis (Sanny BIA1012-8s, São Paulo, Brazil) were performed. In addition, blood samples were collected, and indirect calorimetry was conducted (MetaCheck, Korr, Salt Lake City, UT, USA).

On the day prior to each intervention, participants were instructed to undergo a 12 h overnight fast (starting at 8:00 p.m.), to abstain from alcoholic and energy drinks, and to avoid engaging in intense physical exercise. On each intervention day, fasting blood samples were collected and participants completed questionnaires related to other outcomes of the parent protocol. Breakfast and lunch corresponding to the assigned experimental condition were provided on each intervention day. Meals were consumed at a fixed 4 h interval. Blood samples were collected before breakfast (fasting), 240 min after breakfast, and 240 min after lunch. Thus, the protocol included two independent late postprandial assessments performed after meals with the same experimental characteristics (degree of processing and energy density). During the postprandial period, participants were instructed not to consume any food or energy-containing beverages other than those provided by the study. To ensure compliance with this restriction, capillary blood glucose was measured before each meal, using a cutoff point of 126 mg/dL [25]. In addition, for female participants, menstrual cycle regularity was verified to minimize potential hormonal variations that could influence appetite and eating behavior. The test conditions were performed between days 5 and 21 of the menstrual cycle [26].

### 2.6. Blood Sample Collection

Blood samples (10 mL per collection point) were collected at three time points on each intervention day: (1) after a 12 h overnight fast; (2) 240 min after breakfast consumption; and (3) 240 min after lunch consumption. Blood collections were performed by a trained professional via venipuncture of the antecubital fossa, using EDTA tubes (Vacuette K3E, Greiner Bio-One, Kremsmünster, Austria) and serum separator tubes (Vacuette CAT Serum Sep Clot Activator, Greiner Bio-One, Kremsmünster, Austria). Samples were centrifuged at 3000 rpm at 4 °C for 15 min. Serum was stored and sent to an accredited laboratory responsible for the analysis of glucose, insulin, and triglycerides. Plasma samples for ghrelin and leptin measurements were stored in an ultra-freezer at −80 °C until subsequent analysis.

### 2.7. Hormonal Assays

Plasma leptin concentrations were quantified using an enzyme-linked immunosorbent assay (ELISA) kit (nº. cat. SEA084Hu, Wuhan USCN Business Co., Ltd., Houston, TX, USA), based on a sandwich-type enzyme immunoassay, strictly following the manufacturer’s instructions. Ghrelin concentrations in the samples were determined using a commercial ELISA kit (nº. cat. UCEA991Hu, Wuhan USCN Business Co., Ltd., Houston, TX, USA) based on a competitive inhibition enzyme immunoassay technique, with a lower limit of detection of 44.5 pg/mL. The optical density (OD) of the samples was measured by spectrophotometry using a Synergy HTX microplate reader (serial number 23092022) at 450 nm. Hormone concentrations were calculated from sample OD values by comparison with a previously established standard curve.

### 2.8. Complementary Variables

Data regarding sex, age, education level, smoking status, presence of chronic diseases, and chronic use of medications were collected through a simple and objective questionnaire developed and previously tested by the research group. Economic class was assessed using the Brazilian Economic Classification Criterion (CCEB) [27].

Anthropometric measurements were determined without shoes, with participants wearing light clothing and no heavy accessories. Body weight (kg) was measured using a digital scale (Balmak^®^ BK-50, São Paulo, Brazil), with a capacity of 150 kg and precision of 50 g. Height (m) was measured using a portable stadiometer (Balmak^®^ EST-221, São Paulo, Brazil), with a maximum height of 2.2 m and precision of 1 mm. Waist circumference (cm) was measured at the midpoint between the last rib and the anterior superior iliac crest using a non-elastic measuring tape. With the participant in the anatomical position, the researcher instructed them to take a deep breath, and the measurement was taken at the end of expiration.

Body composition was estimated using segmented octapolar electrical bioimpedance (Sanny BIA1012-8s, São Paulo, Brazil). The assessment was conducted in a private environment, in the presence of two researchers accompanying the participant, who was instructed to remain in the anatomical position, wearing light clothing, and without metallic accessories. Eight electrodes were placed on the right and left hemibodies (wrist, hand, ankle, and foot) [28]. Resistance and reactance data were analyzed using BioSannyX software (version 1.0.4), allowing determination of the following parameters: body fat percentage and body fat-free mass (kg), percentage of total body water, and BMI.

For the measurement of resting energy expenditure (REE), participants were instructed not to engage in vigorous physical exercise on the previous day, not to consume caffeine within the last 24 h, and to fast for 12 h. Before the procedure, axillary temperature (Techline, São Paulo, Brazil) and blood pressure and heart rate (sphygmomanometer, HEM-6122, OMRON, Kyoto, Japan) were measured. REE was assessed by indirect calorimetry (MetaCheck, KoRR, Salt Lake City, UT, USA), in a quiet environment with low lighting and controlled temperature. Participants were instructed to wear a nose clip and breathe through a mouthpiece, ensuring that all expired air passed through the analyzer. Measurements were performed for 11 min, with the first minute being disregarded [29]. REE was calculated using the Weir equation [30] and contributed to the estimation of total energy expenditure (TEE).

Body acceleration was monitored using triaxial accelerometers (ActiGraph wGT3X-BT, ActiGraph LLC, Pensacola, FL, USA) for 5 to 7 days at baseline, positioned on the right hip, and removed only for water-based activities. Participants recorded sleep times, device removal periods, and physical activity practice in a logbook provided on the day of screening. After the device was returned, the data were transferred to ActiLife software (v.6.13.6) for analysis.

### 2.9. Sample Size

This study involves the analysis of pre-specified secondary outcomes from a randomized clinical trial, the primary outcome of which was to evaluate the ad libitum energy intake of individuals subjected to the four experimental conditions described. Consequently, the study was powered for the primary outcome. The sample size was based on the standardized effect size considered “large” (Cohen’s dz = 0.825), found in the study by Hall et al. [8], which measured participants’ energy intake after exposing individuals to diets rich in and free of UPF. The calculation considered the four test conditions: the crossover design used by Hall et al. [8] in the repeated-measures analysis, and a medium effect size (Cohen’s f = 0.25), with two additional test conditions: energy density and degree of processing. Furthermore, since the primary outcome (ad libitum energy intake) shows strong within-subject correlation (r = 0.75), especially in controlled environments [31], an alpha of 5% and a statistical power (1 − β) of 95% were adopted, such that a sample of 19 individuals would be required. The sample size calculation was performed using G*Power software (v. 3.1.97, Heinrich Heine University, Düsseldorf, Germany). All analyses performed in this investigation were considered exploratory, given the secondary nature of these hypotheses.

### 2.10. Statistical Analysis

Linear mixed models were employed to evaluate the effects of experimental conditions over time on metabolic and hormonal outcomes, accounting for the crossover design and repeated measures. Analyses were performed using R software (version 4.5.1, The R Project, Vienna, Austria), with the packages “nlme”, “car”, “emmeans”, “tidyverse”, and “ggplot2”.

The outcomes analyzed included energy substrates (glucose and triglycerides) and hormones (insulin, ghrelin, and leptin). Experimental conditions, meals (240 min post-breakfast and 240 min post-lunch), and their interactions were included in the models as fixed effects. In all models, the fasting measurement corresponding to each outcome was included as an adjustment covariate. Participants’ intercepts were included as random effects in the models. To ensure model adequacy, the assumption of constant variance was tested for each outcome: a homoscedastic model was compared with a model allowing heterogeneous variances across experimental conditions (using the varIdent function), with model selection performed through the Likelihood Ratio Test (LRT), with both models estimated by Maximum Likelihood (ML).

The final selected models were estimated using Restricted Maximum Likelihood (REML), adopting sum-to-zero contrasts for all categorical fixed effects (UPF, ED and Meal). Because each factor had two levels, all main effects and interactions were tested using one numerator degree of freedom (df = 1) and 18 residual degrees of freedom (df = 18). The significance of fixed effects was evaluated through Type III Analysis of Variance (ANOVA). In addition, the estimated marginal means from the full model and from the main effects of the experimental conditions were extracted using the “emmeans” package. A 5% significance level was adopted for all analyses. As the metabolic and hormonal outcomes represented secondary exploratory analyses of the study, no adjustment for multiple comparisons was performed. Consequently, the *p*-values should be interpreted as exploratory.

## 3. Results

### 3.1. Participant Characteristics

**Figure 4:**
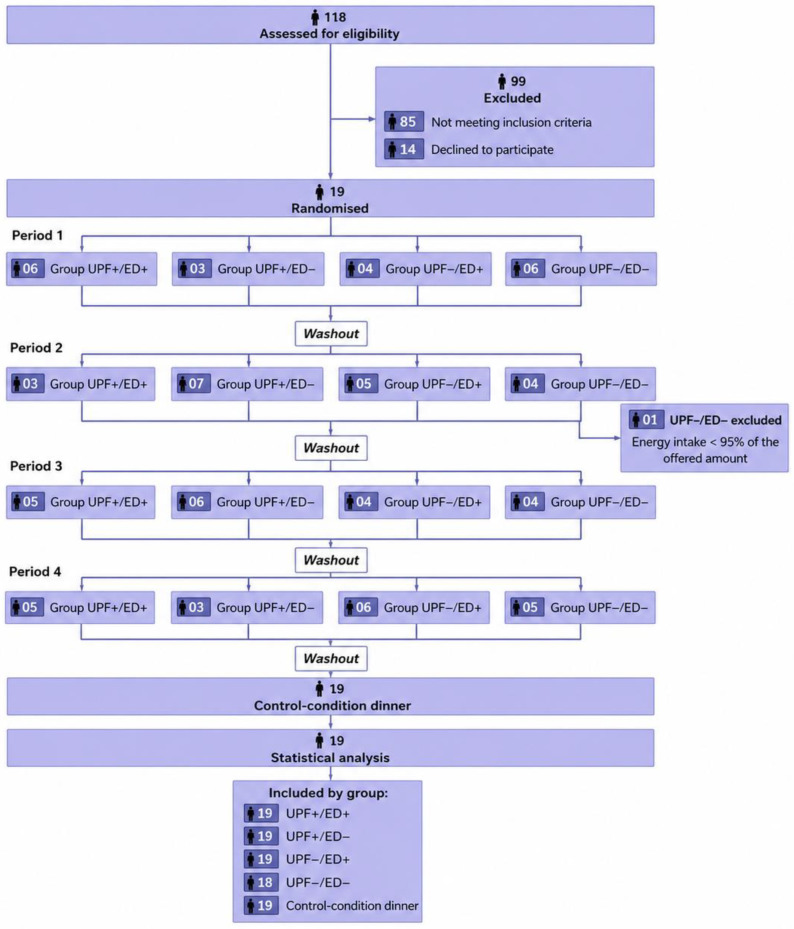
Participant flowchart according to the CONSORT.

Of the 118 screenings conducted, 19 individuals met the eligibility criteria and were included in the study. Of these, one participant was subsequently excluded only from the analyses related to the UPF−/ED− condition for consuming less than 95% of the energy provided in the meals. Figure 4 presents the detailed participant flow.

The sample consisted of young adults, with a mean age of 22.1 (3.2) years, including 10 men (52.6%) and 9 women (47.4%). From a sociodemographic perspective, participants were predominantly classified as belonging to economic classes C1–C2 (47.4%) and self-identified as White or Brown (42.1% for each group). Anthropometric and body composition characteristics showed a mean body weight of 68.3 (11.5) kg and height of 1.70 (0.08) m. Sex-stratified analysis demonstrated that men presented a BMI of 24.3 (2.1) kg/m^2^, waist circumference of 79.5 (4.9) cm, and total body fat mass of 21.2 (4.2)%, whereas women presented a BMI of 22.7 (3.3) kg/m^2^, waist circumference of 71.8 (4.3) cm, and total body fat mass of 33.5 (3.6)%. Regarding fasting metabolic parameters, mean values were 77.6 (8.9) mg/dL for glucose, 59.1 (15) mg/dL for triglycerides, 136.6 (25.2) mg/dL for total cholesterol, and 45.5 (10.2) mg/dL for HDL cholesterol. All descriptive characteristics of the sample are detailed in Table 2.

### 3.2. Cardiometabolic Markers and Appetite-Regulating Hormones

**Figure 5:**
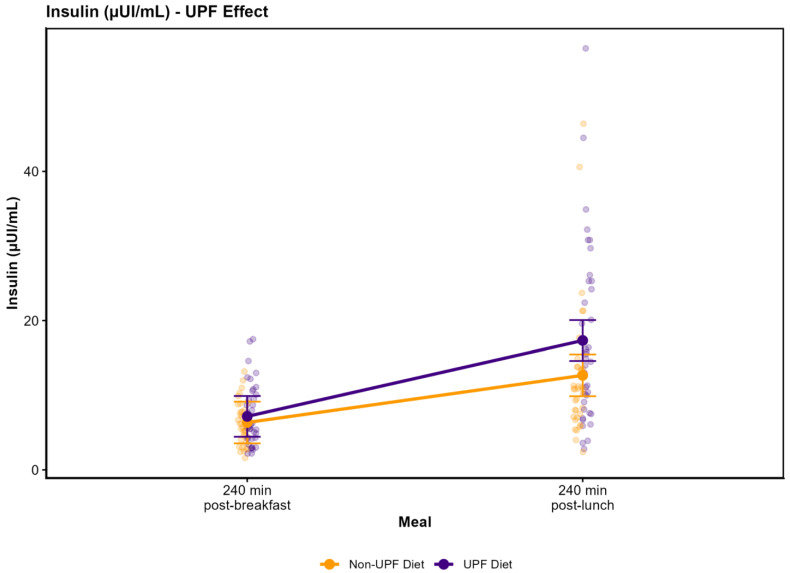
Postprandial insulin (µIU/mL) at 240 min after UPF and non-UPF meals (breakfast and lunch). The bold points represent estimated marginal means specific to each meal obtained from linear mixed models, and the error bars indicate 95% confidence intervals (95% CI). Semi-transparent points represent individual data.

Meal timing (post-breakfast and post-lunch assessments) significantly affected glucose (*p* = 0.034), insulin (*p* < 0.001) and leptin (*p* = 0.003) concentrations. It was found to have significantly higher insulin concentrations under the UPF condition compared with the Non-UPF condition (*p* = 0.016) (Table 3; Figure 5). There was no significant interaction between UPF and meal timing for insulin (*p* = 0.09). Regarding glucose and triglycerides, no significant main effects of UPF (*p* = 0.080 and *p* = 0.199, respectively) or ED (*p* = 0.710 and *p* = 0.141, respectively) were observed, nor were there significant interactions, at any of the postprandial time points (*p* = 0.650 and *p* = 0.817, respectively). No significant main effects for ED were identified, and there was no statistical interaction between UPF and ED for any of the cardiometabolic markers evaluated.

Analyses of appetite-regulating hormones were conducted in subsamples, due to financial constraints. Ghrelin concentrations were evaluated in 13 participants. Leptin was analyzed in only 8 participants, as 5 samples had concentrations below the assay detection limit (<0.058 ng/mL). Analyses did not reveal statistically significant effects of UPF exposure on ghrelin (*p* = 0.235) or leptin (*p* = 0.795) concentrations. Similarly, no significant main effects of ED were observed for ghrelin (*p* = 0.515) or leptin (*p* = 0.994). Furthermore, the interaction between UPF and ED also did not yield significant changes in ghrelin (*p* = 0.989) and leptin (*p* = 0.263) concentrations, and these responses did not interact significantly with meal timing (Table 3). Graphs from all results may be seen in the Supplementary Figures S1–S13.

## 4. Discussion

This study investigated the acute effects of meals rich in or free of UPF, with high or low ED, matched for energy, macronutrients, fiber, saturated fat, free sugar, and sodium, on the concentrations of cardiometabolic markers and appetite-regulating hormones in non-obese adults. The main finding was that the acute consumption of UPF-rich meals resulted in higher circulating insulin concentrations during the late postprandial phase compared to UPF-free meals. However, glucose concentrations did not differ significantly between conditions, suggesting that UPF intake may require delayed postprandial insulin clearance or a prolonged insulin demand to maintain euglycemia, independent of the meals’ ED. Nevertheless, the physiological mechanisms underlying this finding remain to be elucidated.

Generally, the deleterious effects of UPF are attributed to their intrinsically unbalanced nutritional profile, characterized by a high glycemic index, higher fat and added sugar content, and lower dietary fiber content [32]. However, in the present study, the test meals were matched for nutritional composition across experimental groups, and no significant effects of ED or interaction between ED and UPF were observed. The absence of a significant interaction suggests that, under the controlled nutritional conditions of this study, ED did not modify the metabolic response associated with UPF consumption, indicating no evidence of synergistic effects between these two factors. Furthermore, it is unlikely that the observed metabolic responses are explained by variations in macronutrients or fiber, suggesting that the degree of food processing may represent an important determinant of late postprandial circulating insulin, and that it operates independently of ED. This finding supports the hypothesis that structural and technological characteristics of UPF, including degradation of the food matrix, eating rate, and rapid nutrient bioavailability and absorption, directly influence metabolic modulation [33,34]. In addition, interference from non-nutritional components commonly present in UPF, such as cosmetic food additives, may act as metabolic disruptors independently of their nutritional profile, influencing several metabolic pathways, such as alterations in the gut microbiota and enteroendocrine signaling [35,36,37,38,39,40]. Such effects remain to be determined in future studies.

Consistent with our findings, Çelik and Ulug [12] reported a significantly greater total area under the curve (tAUC) for insulin following consumption of a UPF breakfast compared with a non-UPF breakfast in adults with and without obesity. However, unlike the present study, the meals were not matched for energy density, free sugar, or dietary fiber, making it difficult to determine whether the observed effects were attributable to food processing itself or to nutritional differences between conditions. The persistence of an elevated insulin response under tightly matched nutritional conditions in our study strengthens the hypothesis that food processing independently influences acute postprandial glyco-insulinemic regulation. Physiologically, systemic insulin concentrations depend heavily on the fractional hepatic insulin clearance rate, rather than merely reflecting pancreatic secretion [41]. The rapid nutrient bioaccessibility typical of UPFs with a degraded food matrix possibly forces the homeostatic system into an acute adaptation, transiently reducing hepatic insulin clearance and thereby prolonging the half-life of circulating insulin to prevent postprandial hyperglycemia [42,43]. Therefore, the prolonged hyperinsulinemia observed in this late postprandial phase possibly reflects a physiological compensation to maintain euglycemia [41,44]. Nevertheless, these mechanisms remain speculative and should be interpreted as hypotheses requiring confirmation in future studies. It is also worth noting that, despite the absence of a statistically significant UPF × meal interaction (*p* = 0.09), visual inspection of the estimated means suggests that the difference between UPF conditions may have been more pronounced after lunch than after breakfast. One possible explanation is that lunch provided a substantially greater caloric load (40–45% vs. 20–25% of daily energy intake), which would be expected to elicit a larger postprandial insulin response, potentially making differences between UPF conditions more apparent. Alternatively, repeated exposure to the experimental meals throughout the day may also have contributed to amplifying these differences. However, because the UPF × meal interaction was not statistically significant, these interpretations remain speculative.

Regarding appetite regulation, neither ghrelin nor leptin concentrations differed significantly between the intervention conditions. These findings are consistent with previous controlled feeding studies reporting limited or absent effects of acute UPF exposure on classical appetite-related hormones. Hall et al. [8] found no differences in fasting ghrelin concentrations after 14 days of UPF consumption, while Galdino-Silva et al. [7] observed no significant acute changes in glucose, insulin, ghrelin, or leptin after consumption of a UPF meal compared with a non-UPF meal. Similarly, Martins et al. [45] reported no significant changes in leptin concentrations following increased UPF intake in adolescents. The absence of detectable hormonal differences in the present study may be related to the participants’ metabolically healthy profile and the extensive matching of the test meals, which may have minimized differences in classical homeostatic pathways. It should also be considered that, in the present study, such hormone analyses were conducted in reduced subsamples due to financial constraints, particularly for leptin, which 5 samples were below the detection range of the assay, limiting statistical power to detect small differences. Importantly, these findings do not exclude the possibility that UPF influences eating behavior through mechanisms not assessed in this study. Given the substantial interindividual variability in postprandial peptide responses, acute metabolic effects of UPF may occur independently of detectable changes in ghrelin or leptin concentrations [46].

There are some limitations to consider. The study included healthy, non-obese young adults. Due to the specific characteristics of the study sample, caution should be taken when extrapolating these results beyond this specific population. In addition, although all female participants were assessed within the predefined study window (days 5–21 of the menstrual cycle), the exact menstrual cycle day or phase at each intervention visit was not recorded. Therefore, we were unable to evaluate whether menstrual cycle phases were balanced across the crossover conditions, and a potential influence of hormonal fluctuations on metabolic responses cannot be completely excluded. Measurements were performed 4 h after meal ingestion, and different results might have been observed if multiple postprandial measurements had been conducted to capture dynamic fluctuations. Because blood collections were restricted solely to 240 min post-meal, the study missed the dynamic peak and early metabolic excursions typical of healthy individuals. This limited the interpretation of early-phase insulin kinetics, which would have required serial postprandial measurements (30, 60, 90 and 120 min) to track such dynamic fluctuations, thereby helping to differentiate whether the observed values reflect higher insulin secretion or slower clearance. Furthermore, our study was limited to the investigation of classical hormones; however, measuring additional hormones could provide further insight into the impact of meals on postprandial hormonal responses. In addition, the postprandial period is characterized by complex physiological dynamics, with rapid fluctuations in glucose, insulin, and other regulatory metabolites, which limit the extrapolation of the results. The sample size was calculated for the primary outcome of the study, ad libitum food intake, thereby limiting statistical power for the secondary metabolic and hormonal outcomes investigated here. Additionally, hormonal analyses of ghrelin and leptin were conducted in a subgroup of 13 participants due to financial constraints on laboratory testing. Finally, five losses were due to the leptin assay’s detection limit. Furthermore, no comparison was made between participants with detectable and undetectable leptin concentrations. Therefore, the possibility that the subgroup included in the analyses differed in characteristics relevant to leptin physiology cannot be ruled out. Accordingly, the overall findings should be considered exploratory, and the ghrelin and leptin results, in particular, should be interpreted with due caution.

## 5. Conclusions

The results indicate that the acute consumption of meals rich in UPF raises circulating insulin concentrations during the late postprandial period compared to meals free of UPF. Given that the experimental diets were strictly matched for macronutrients, fiber, saturated fat, free sugar, and sodium, the level of processing appears to be a determining factor in the response; however, these findings should be interpreted with caution. This prolonged hyperinsulinemia, accompanied by the maintenance of euglycemia, may reflect a physiological adaptation, such as delayed postprandial insulin clearance or a sustained demand for insulin, in the metabolism of foods with a degraded structural matrix. These findings reinforce the importance of considering the degree of food processing and food matrix integrity as a relevant determinant of acute metabolic responses. No differences were observed in circulating ghrelin or leptin concentrations. The absence of changes in classical appetite hormones highlights the complexity of appetite regulation. Future, longer-term studies employing standardized mixed-meal tolerance tests with frequent serial blood sampling are needed to precisely elucidate insulin kinetic mechanisms and their potential long-term implications.

## Figures and Tables

**Table 1:** Nutritional composition of meals provided for individuals with a total energy value of 2000 kcal.

Meal	ED (kcal/g)	g	Kcal	CHO (g)	Protein (g)	Fat (g)	Free Sugar (g)	Sat. Fat (g)	Fiber (g)	Na (mg)	UPF (kcal)	UPF (%)
Breakfast												
UPF+/ED+	2.16	204.10	442.00	57.00	22.10	13.60	8.74	4.90	7.16	690.70	356.00	80.54
UPF+/ED−	1.16	401.40	467.00	62.70	22.40	13.60	8.62	4.90	6.68	670.50	376.00	80.51
UPF−/ED+	2.23	205.60	460.00	63.00	23.90	13.90	9.14	4.50	7.40	679.00	0.00	0.00
UPF−/ED−	1.20	393.20	474.00	61.70	27.60	13.70	9.34	4.60	6.70	661.70	0.00	0.00
Lunch												
UPF+/ED+	2.00	409.00	822.00	109.80	40.30	24.20	23.44	9.50	9.70	1824.50	686.00	84.74
UPF+/ED−	1.09	786.50	862.00	114.20	39.50	27.00	22.82	9.50	10.48	1849.60	691.00	80.16
UPF−/ED+	2.10	409.50	860.00	119.10	37.00	25.70	22.76	9.20	9.10	1759.60	0.00	0.00
UPF−/ED−	1.03	775.00	805.00	110.70	39.70	24.30	22.28	9.50	9.90	1778.40	0.00	0.00

CHO: carbohydrates; Sat. fat: Saturated fat; UPF: Ultra-processed foods; ED: Energy density; g: Grams; kcal: Kilocalories; UPF (kcal): Kilocalories derived from ultra-processed foods; UPF (%): Percentage of ultra-processed foods per meal.

**Table 2:** Descriptive characteristics of the sample (n = 19).

Variables	Men (n = 10)		Women (n = 9)		Total (n = 19)	
	n	%	n	%	n	%
Skin color						
Black	2	20.00	1	11.10	3	15.80
White	6	60.00	2	22.20	8	42.10
Brown	2	20.00	6	66.70	8	42.10
Economic classification ^a^						
A	3	30.00	0	0.00	3	15.80
B1–B2	3	30.00	2	22.20	5	26.30
C1–C2	3	30.00	6	66.70	9	47.40
D–E	1	10.00	1	11.10	2	10.50
	Mean	SD	Mean	SD	Mean	SD
Age (years)	22.50	3.72	21.80	2.59	22.15	3.16
Weight (kg)	75.60	6.57	60.20	10.40	68.29	11.48
Height (m)	1.76	0.04	1.63	0.05	1.69	0.08
WC (cm)	79.50	4.86	71.80	4.28	75.80	5.96
BMI (kg/m^2^)	24.30	2.12	22.70	3.27	23.50	2.70
TFM (%)	21.20	4.24	33.50	3.61	27.05	7.39
SMM (%)	44.20	3.12	32.60	3.31	38.69	6.69
Total body water (%)	57.70	3.09	47.50	3.61	52.88	6.17
REE (kcal)	1450.00	231.40	1022.20	113.00	1247.36	283.90
CPM	544.60	207.70	466.90	112.50	507.81	169.67
MET	1.57	0.17	1.49	0.10	1.53	0.14
MVPA time (min)	58.40	28.50	41.70	20.50	50.48	25.79
TEE (kcal)	2207.20	574.90	1513.00	124.70	1878.39	546.81
Glucose (mg/dL)	74.20	10.70	81.40	4.03	77.63	8.86
Triglycerides (mg/dL)	60.80	17.40	57.30	12.70	59.15	15.03
Total cholesterol (mg/dL)	135.40	30.90	138.00	18.60	136.63	25.17
HDL cholesterol (mg/dL)	42.20	11.40	49.20	7.68	45.52	10.19

WC: Waist circumference; BMI: Body mass index; TFM: Total fat mass; SMM: Skeletal muscle mass; REE: Resting energy expenditure; CPM: Counts per minute; MET: Metabolic equivalent of task; MVPA: Moderate-to-vigorous physical activity; TEE: Total energy expenditure; SD: Standard deviation; ᵃ Defined according to the Brazilian Economic Classification Criterion (CCEB).

**Table 3:** Estimated marginal means of cardiometabolic markers under UPF and ED conditions obtained from linear mixed models adjusted for baseline values (n = 19).

Outcomes	Post Meals (240 min)	UPF		Non-UPF		Meal	UPF	ED	UPF × ED
		Low ED	High ED	Low ED	High ED	*p*-Value ^a^	*p*-Value ^b^	*p*-Value ^c^	*p*-Value ^d^
		Mean (95% CI)	Mean (95% CI)	Mean (95% CI)	Mean (95% CI)				
Glucose (mg/dL)	breakfast	83.4 (79.1–87.7)	80.7 (77.1–84.4)	83.2 (80.5–86)	82.3 (78.5–86.2)	0.034	0.080	0.710	0.699
	lunch	82.6 (78.3–86.9)	83.7 (80.0–87.3)	85.7 (83–88.5)	86.6 (82.8–90.5)				
Triglycerides (mg/dL)	breakfast	90.3 (76.4–104.1)	81.9 (71.8–92.1)	86.3 (75–97.6)	82.2 (72.6–91.7)	0.430	0.199	0.141	0.559
	lunch	90.6 (76.8–104.5)	88.4 (78.2–98.6)	85.0 (73.7–96.3)	84.6 (75.1–94.2)				
Insulin (µUI/mL)	breakfast	7.7 (4.1–11.4)	6.55 (2.9–10.1)	6.3 (2.5–10.1)	6.3 (2.7–9.9)	<0.001	0.016	0.621	0.721
	lunch	17.6 (14–21.3)	16.9 (13.3–20.5)	12.8 (9–16.6)	12.4 (8.8–16.1)				
Ghrelin (pg/mL)	breakfast	236 (100.5–372)	244 (107.6–380)	213 (71.8–354)	247 (111.3–384)	0.294	0.235	0.515	0.989
	lunch	299 (162.7–435)	346 (210–482)	225 (89.3–361)	243 (107.2–380)				
Leptin (ng/mL) ^e^	breakfast	7.6 (5.6–9.6)	7.2 (5.1–9.2)	7.2 (5.2–9.3)	7.60 (5.5–9.6)	0.003	0.795	0.994	0.263
	lunch	9.2 (7.2–11.2)	8.4 (6.4–10.5)	8.7 (6.6–10.7)	9.5 (7.5–11.5)				

UPF: ultra-processed foods; ED: energy density; 95% CI: 95% confidence interval. ᵃ: *p*-value for the main effects of the meal (post-breakfast vs. post -lunch). ᵇ: *p*-value for the main effects of processing level (UPF vs. non-UPF); ᶜ: *p*-value for the main effects of ED (Low ED vs. High ED). ^d^: *p*-value for the UPF*ED interaction. ^e^: analysis based on 8 subjects (96 samples).

## Data Availability

The data presented in this study are openly available in [OSF] at [https://osf.io/j9kup/overview?view_only=769dcd1a3eb0491a8a6afe9515917f68] accessed on 17 June 2026.

## Supplementary Materials

The following supporting information can be downloaded at: https://www.mdpi.com/article/10.3390/nu18152534/s1, Figure S1: Postprandial glucose (mg/dL) at 240 min after UPF and non-UPF meals; Figure S2: Postprandial glucose (mg/dL) at 240 min after low ED and high ED meals; Figure S3: Postprandial glucose (mg/dL) at 240 min after UPF and ED interaction meals; Figure S4: Postprandial triglycerides (mg/dL) at 240 min after UPF and non-UPF meals; Figure S5: Postprandial triglycerides (mg/dL) at 240 min after low ED and high ED meals; Figure S6: Postprandial triglycerides (mg/dL) at 240 min after UPF and ED interaction meals; Figure S7: Postprandial insulin (mg/dL) at 240 min after low ED and high ED meals; Figure S8: Postprandial insulin (mg/dL) at 240 min after UPF and ED interaction meals; Figure S9: Postprandial ghrelin (mg/dL) at 240 min after UPF and non-UPF meals; Figure S10: Postprandial ghrelin (mg/dL) at 240 min after low ED and high ED meals; Figure S11: Postprandial ghrelin (mg/dL) at 240 min after UPF and ED interaction meals; Figure S12: Postprandial leptin (mg/dL) at 240 min after UPF and non-UPF meals; Figure S13: Postprandial leptin (mg/dL) at 240 min after low ED and high ED meals; Figure S14: Postprandial leptin (mg/dL) at 240 min after UPF and ED interaction meals; Table S1: Checklist of items to be included when reporting randomized crossover clinical trials (CONSORT).
