# Supplementary material for: Effects of Ultra-Processed Meals with Differing Energy Density on Postprandial Glyco-Insulinemic and Appetite-Related Hormonal Responses: A Randomized Crossover Trial
Source: Nutrients. 2026 Aug 4;18(15):2534. doi: 10.3390/nu18152534 (PMC13468057; doi:10.3390/nu18152534)
Supplement: Supplementary file 1 [file nutrients-18-02534-s001.zip › nutrients-4433826-supplementary.pdf]

Effects of Ultra-Processed Meals with Differing Energy Density on Postprandial Glyco-Insulinemic and Appetite-Related Hormonal Responses: A Randomized Crossover Trial

Isabelle Rodrigues de Souza Gama [1,2], Déborah Tenório da Costa Paula [1,3], Delis Barbosa Soares [1,2], Micnéias Róberth Pereira [1,2], Ludmila de Melo Barros [1,2], Guilherme César Oliveira de Carvalho [1,2], Vanessa Amorim Peixoto [1,2], Débora Cavalcante Ferro [1,2], Marianna Victoria Cerqueira Rocha [1,2], Cellyne Veronica da Silva [1], Priscila Adelina Alves Viana [1], Rodrigo Tenório Lins Carnaúba [1], Thayná Xavier Rocha de Oliveira [1], José Israel Rodrigues Júnior [2], Mateus de Lima Macena [1], André Eduardo da Silva-Júnior [1], Fabiana Andréa Moura [2] and Nassib Bezerra Bueno [1,2,3,*]

[1] Laboratório de Nutrição e Metabolismo (LANUM), Faculdade de Nutrição (FANUT), Universidade Federal de Alagoas, Maceió 57072-900, AL, Brazil
[2] Postgraduate Program in Nutrition, Faculdade de Nutrição, Universidade Federal de Alagoas, Maceió 57072-900, AL, Brazil
[3] Postgraduate Program in Nutrition, Escola Paulista de Medicina, Universidade Federal de São Paulo, São Paulo 04023-062, SP, Brazil
* Correspondence: nassib.bueno@fanut.ufal.br; Tel.: +55-82-99976-6895

Supplementary Material

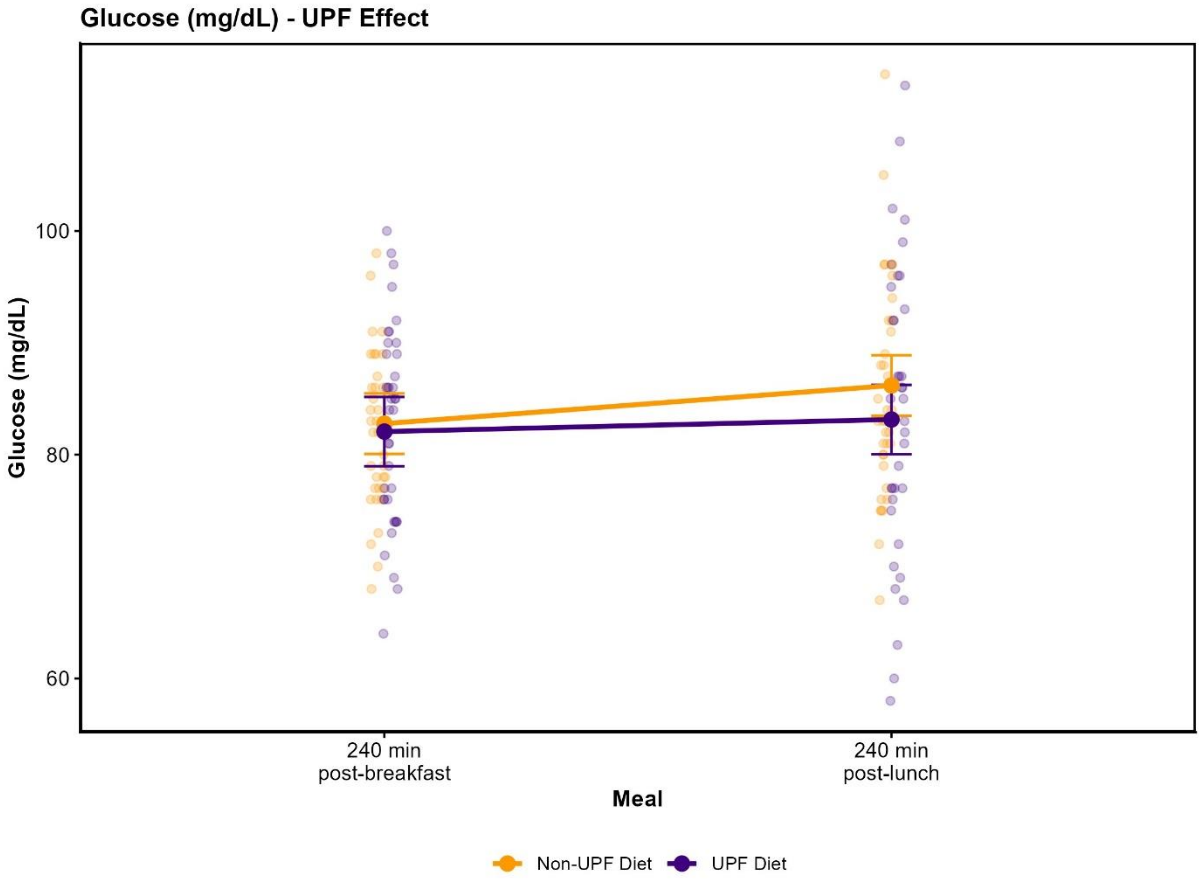

Supplementary Figure S1 Postprandial glucose (mg/dL) at 240 min after UPF and non-UPF meals (breakfast and lunch). The bold points represent estimated marginal means obtained from linear mixed models, and the error bars indicate 95% confidence intervals (95% CI). Semi-transparent points represent individual data.

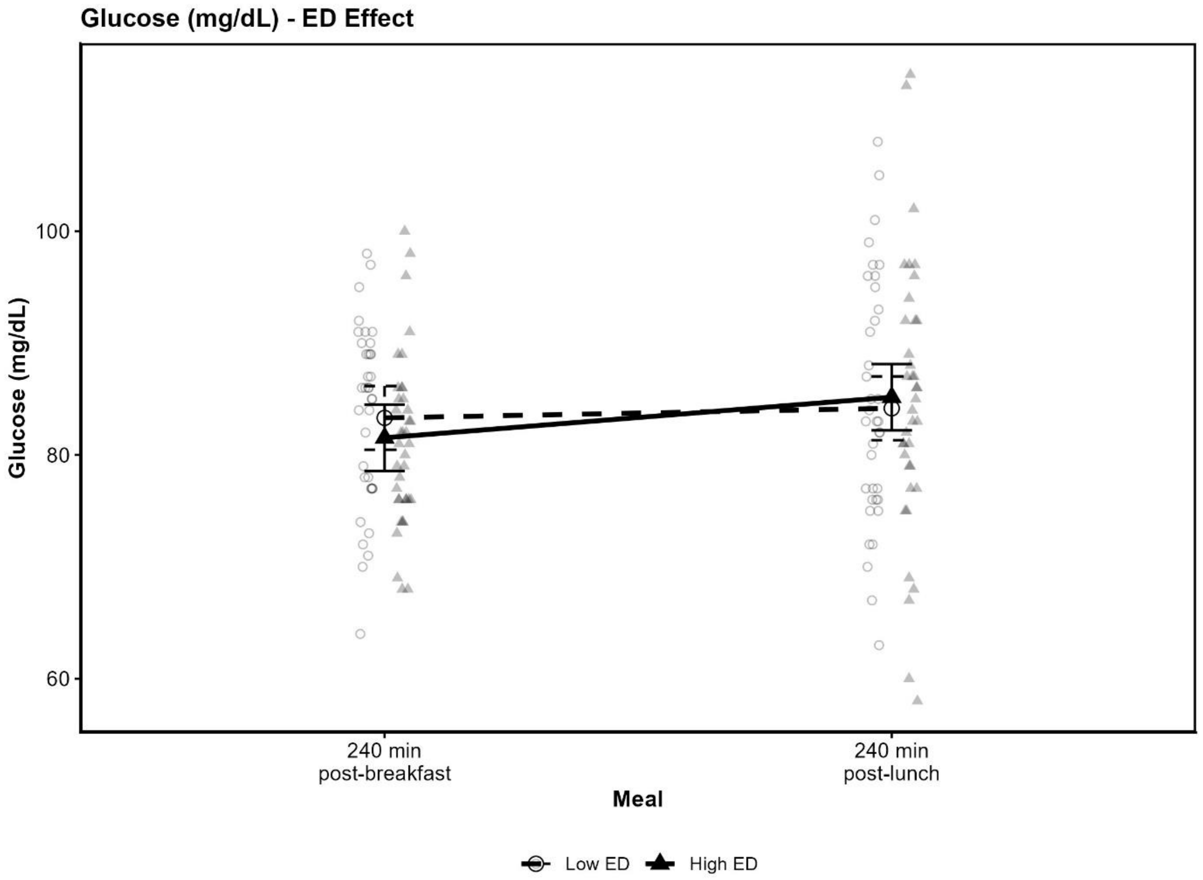

Supplementary Figure S2 Postprandial glucose (mg/dL) at 240 min after low ED and high ED meals (breakfast and lunch). The bold points represent estimated marginal means obtained from linear mixed models, and the error bars indicate 95% confidence intervals (95% CI). Semi-transparent points represent individual data.

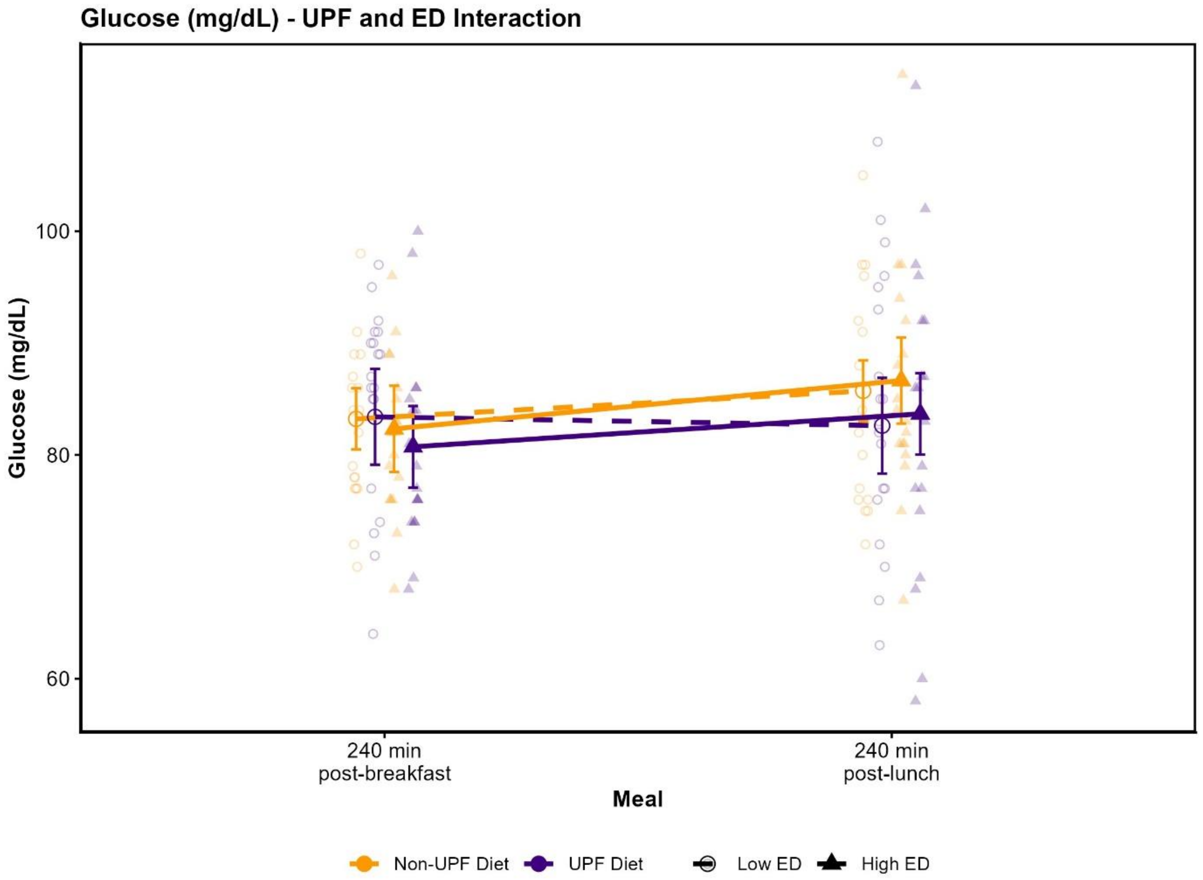

Supplementary Figure S3 Postprandial glucose (mg/dL) at 240 min after UPF and ED interaction meals (breakfast and lunch). The bold points represent estimated marginal means obtained from linear mixed models, and the error bars indicate 95% confidence intervals (95% CI). Semi-transparent points represent individual data.

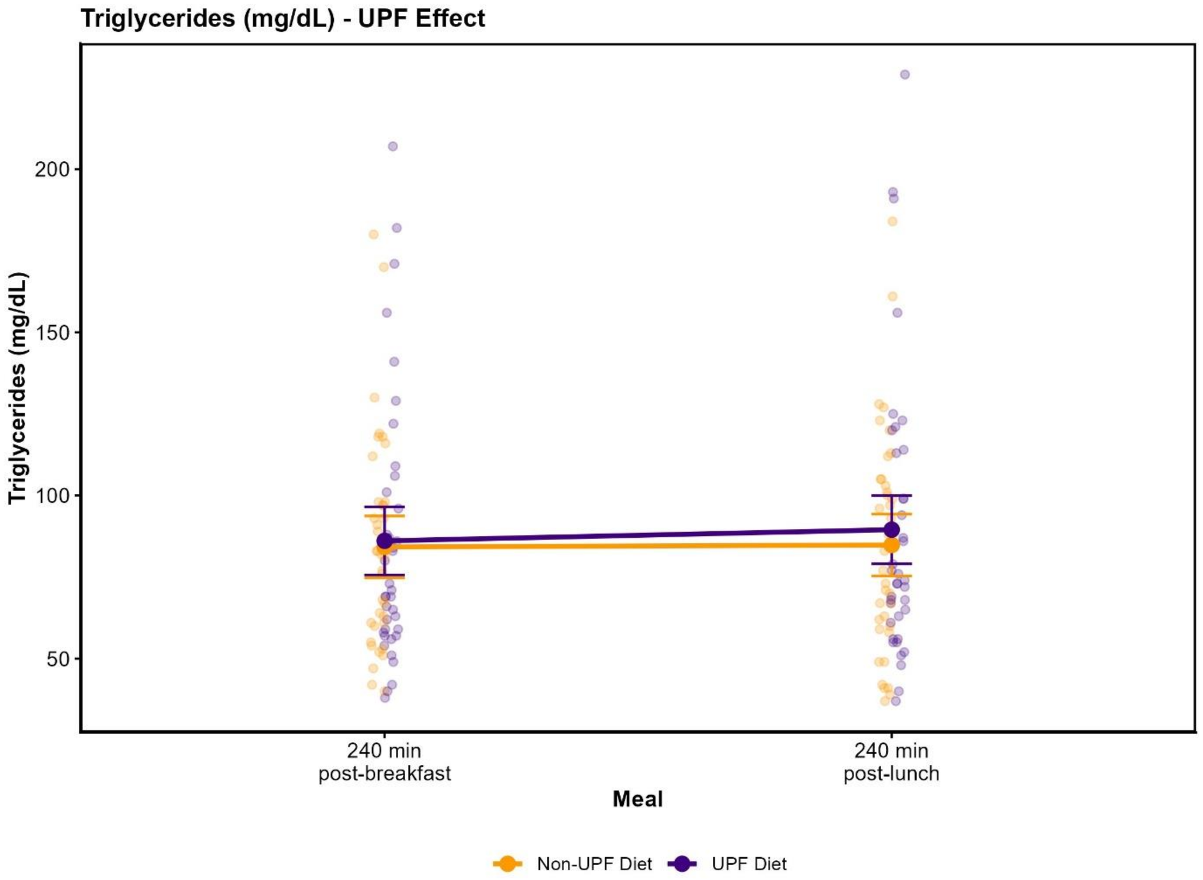

Supplementary Figure S4 Postprandial triglycerides (mg/dL) at 240 min after UPF and non-UPF meals (breakfast and lunch). The bold points represent estimated marginal means obtained from linear mixed models, and the error bars indicate 95% confidence intervals (95% CI). Semi-transparent points represent individual data.

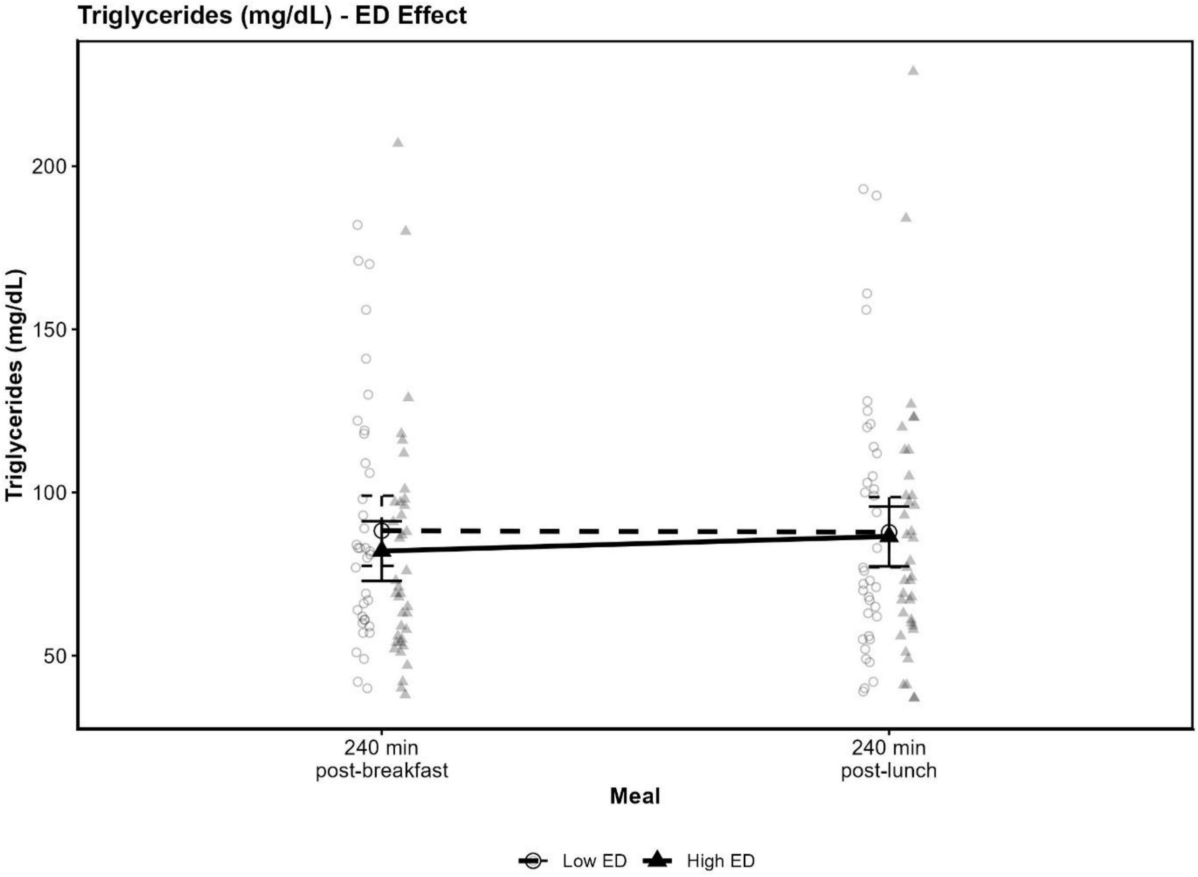

Supplementary Figure S5 Postprandial triglycerides (mg/dL) at 240 min after low ED and high ED meals (breakfast and lunch). The bold points represent estimated marginal means obtained from linear mixed models, and the error bars indicate 95% confidence intervals (95% CI). Semi-transparent points represent individual data.

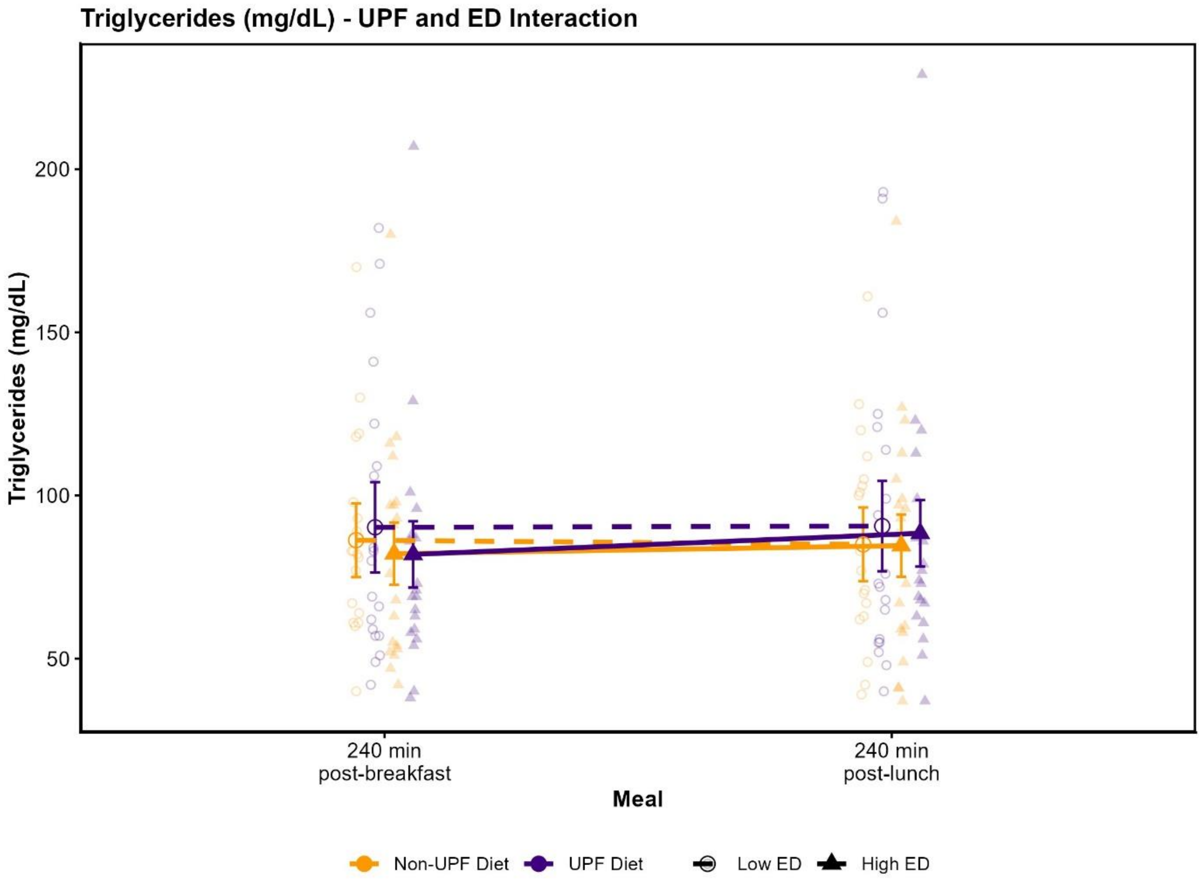

Supplementary Figure S6 Postprandial triglycerides (mg/dL) at 240 min after UPF and ED interaction meals (breakfast and lunch). The bold points represent estimated marginal means obtained from linear mixed models, and the error bars indicate 95% confidence intervals (95% CI). Semi-transparent points represent individual data.

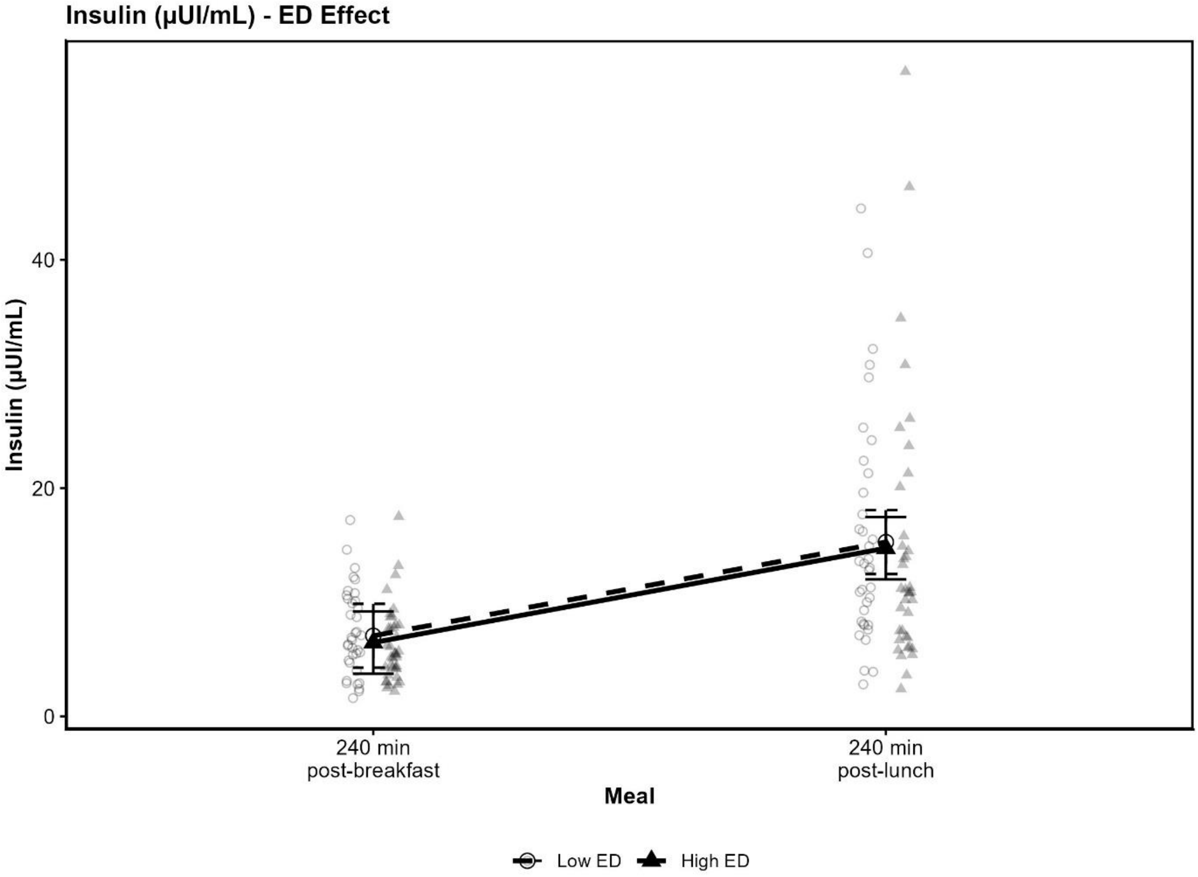

Supplementary Figure S7 Postprandial insulin (mg/dL) at 240 min after low ED and high ED meals (breakfast and lunch). The bold points represent estimated marginal means obtained from linear mixed models, and the error bars indicate 95% confidence intervals (95% CI). Semi-transparent points represent individual data.

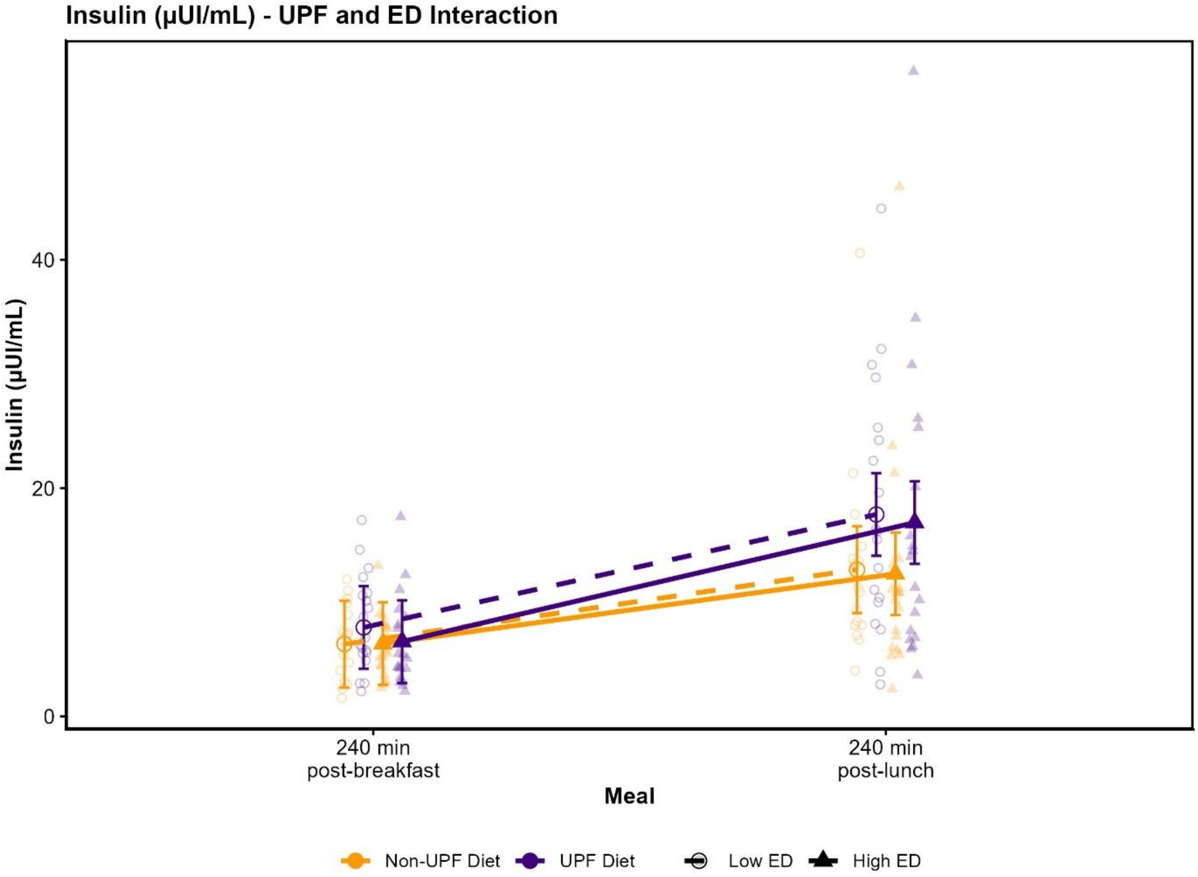

Supplementary Figure S8 Postprandial insulin (mg/dL) at 240 min after UPF and ED interaction meals (breakfast and lunch). The bold points represent estimated marginal means obtained from linear mixed models, and the error bars indicate 95% confidence intervals (95% CI). Semi-transparent points represent individual data.

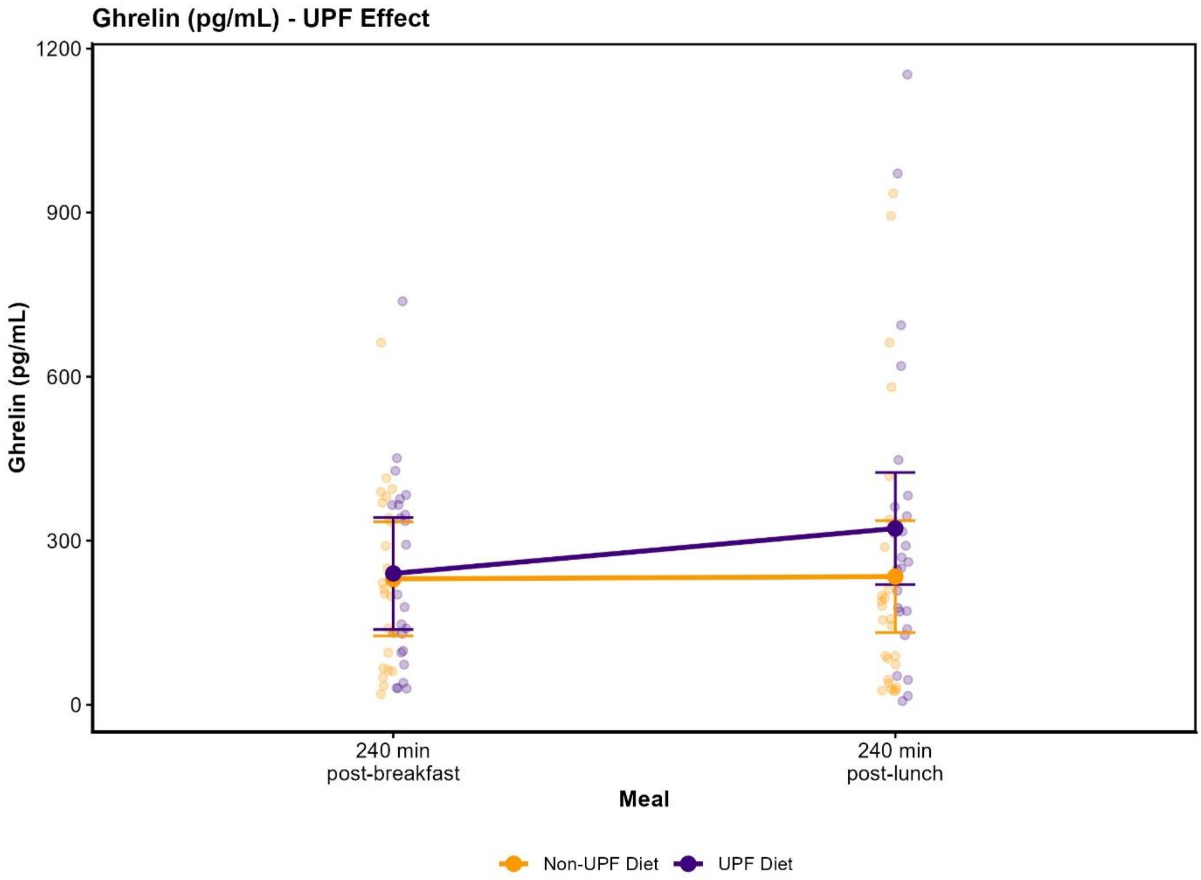

Supplementary Figure S9 Postprandial ghrelin (mg/dL) at 240 min after UPF and non-UPF meals (breakfast and lunch). The bold points represent estimated marginal means obtained from linear mixed models, and the error bars indicate 95% confidence intervals (95% CI). Semi-transparent points represent individual data.

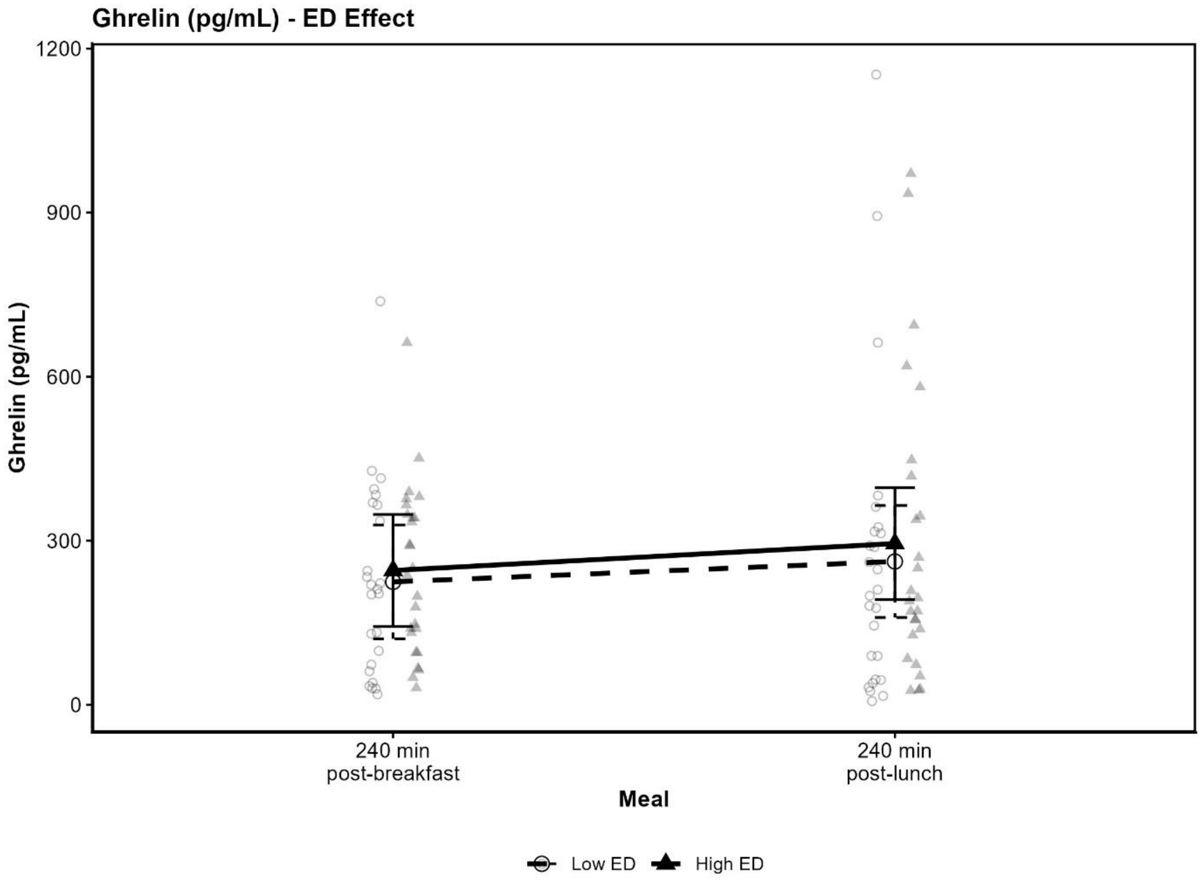

Supplementary Figure S10 Postprandial ghrelin (mg/dL) at 240 min after low ED and high ED meals (breakfast and lunch). The bold points represent estimated marginal means obtained from linear mixed models, and the error bars indicate 95% confidence intervals (95% CI). Semi-transparent points represent individual data.

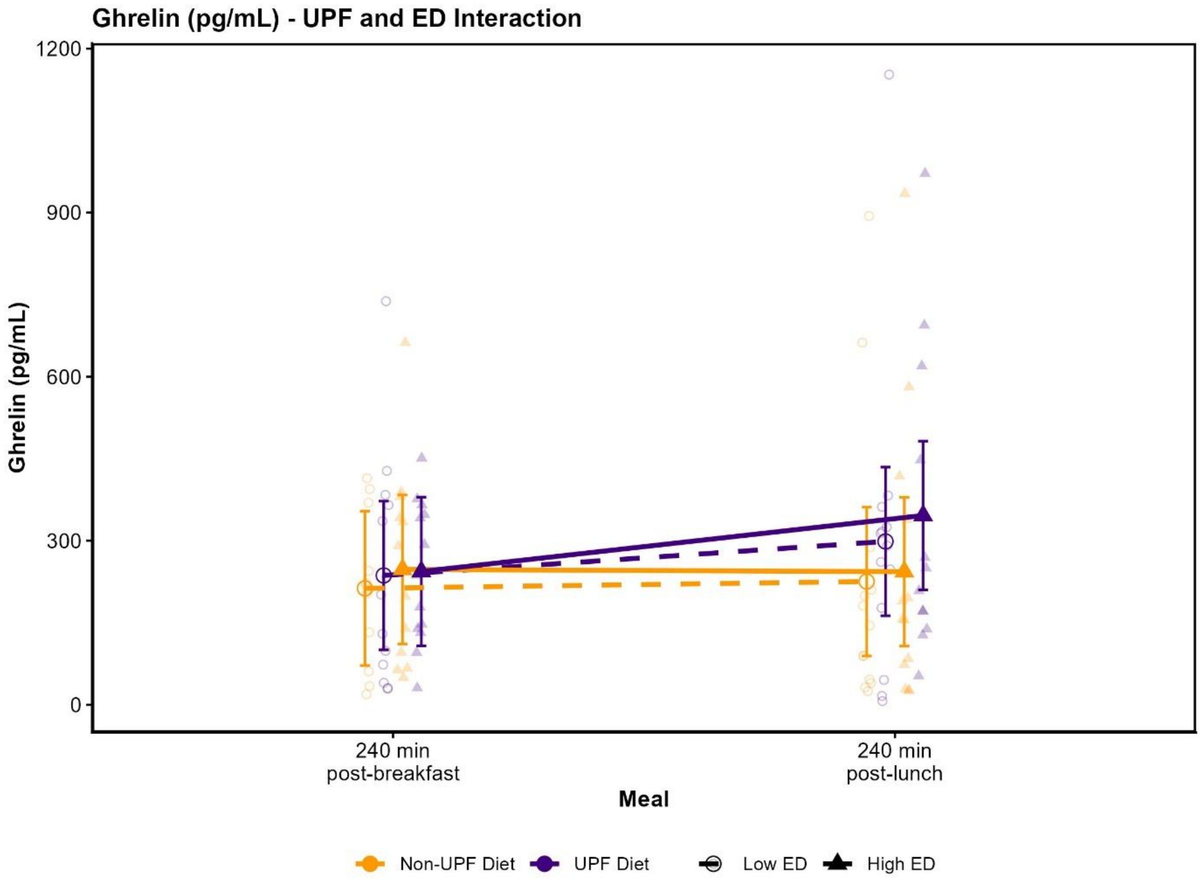

Supplementary Figure S11 Postprandial ghrelin (mg/dL) at 240 min after UPF and ED interaction meals (breakfast and lunch). The bold points represent estimated marginal means obtained from linear mixed models, and the error bars indicate 95% confidence intervals (95% CI). Semi-transparent points represent individual data.

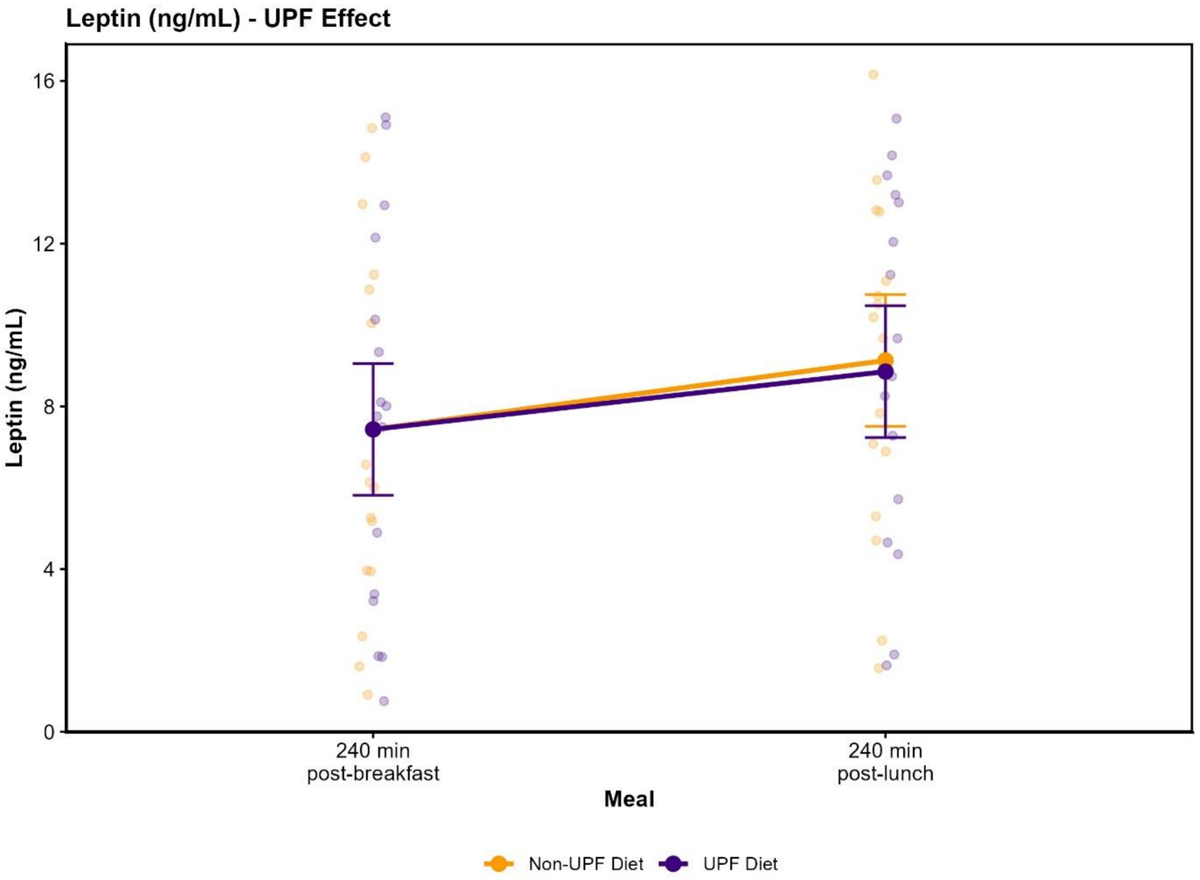

Supplementary Figure S12 Postprandial leptin (mg/dL) at 240 min after UPF and non-UPF meals (breakfast and lunch). The bold points represent estimated marginal means obtained from linear mixed models, and the error bars indicate 95% confidence intervals (95% CI). Semi-transparent points represent individual data.

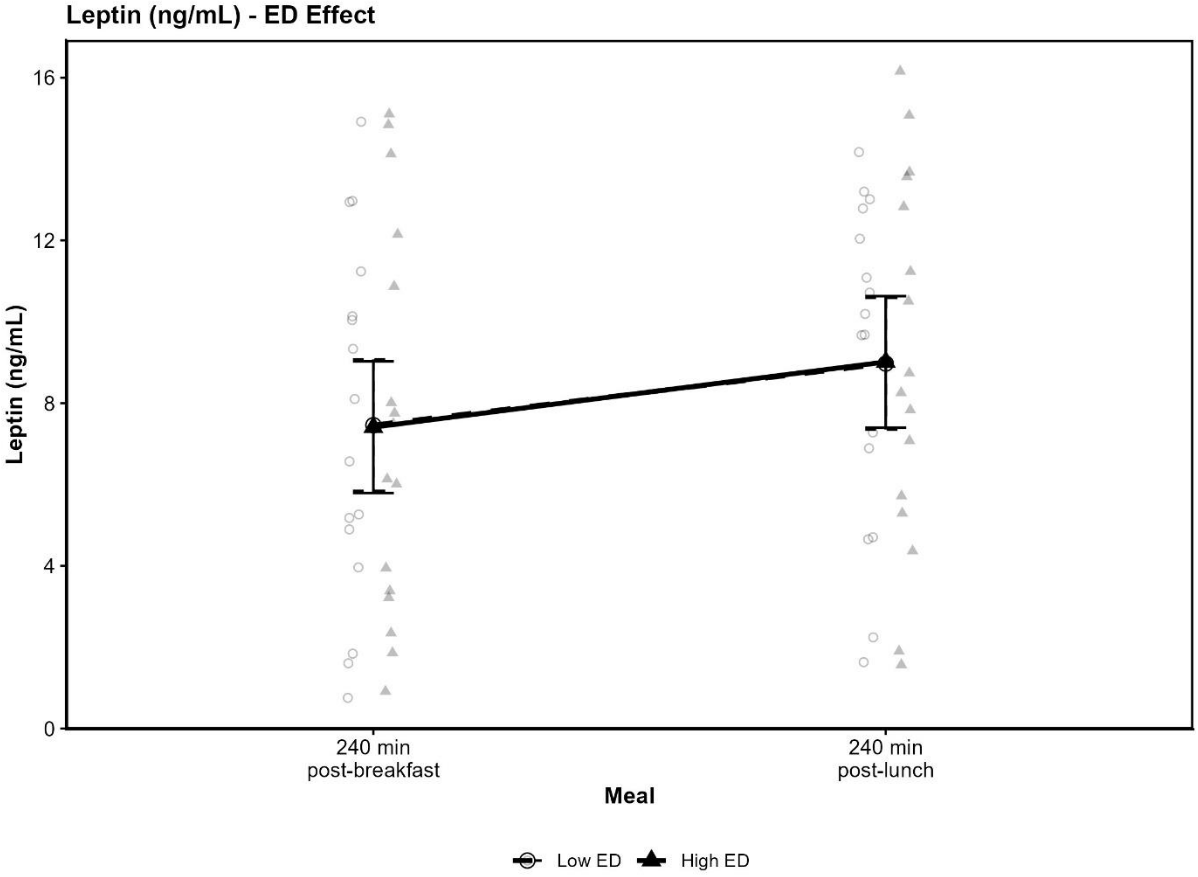

Supplementary Figure S13 Postprandial leptin (mg/dL) at 240 min after low ED and high ED meals (breakfast and lunch). The bold points represent estimated marginal means obtained from linear mixed models, and the error bars indicate 95% confidence intervals (95% CI). Semi-transparent points represent individual data.

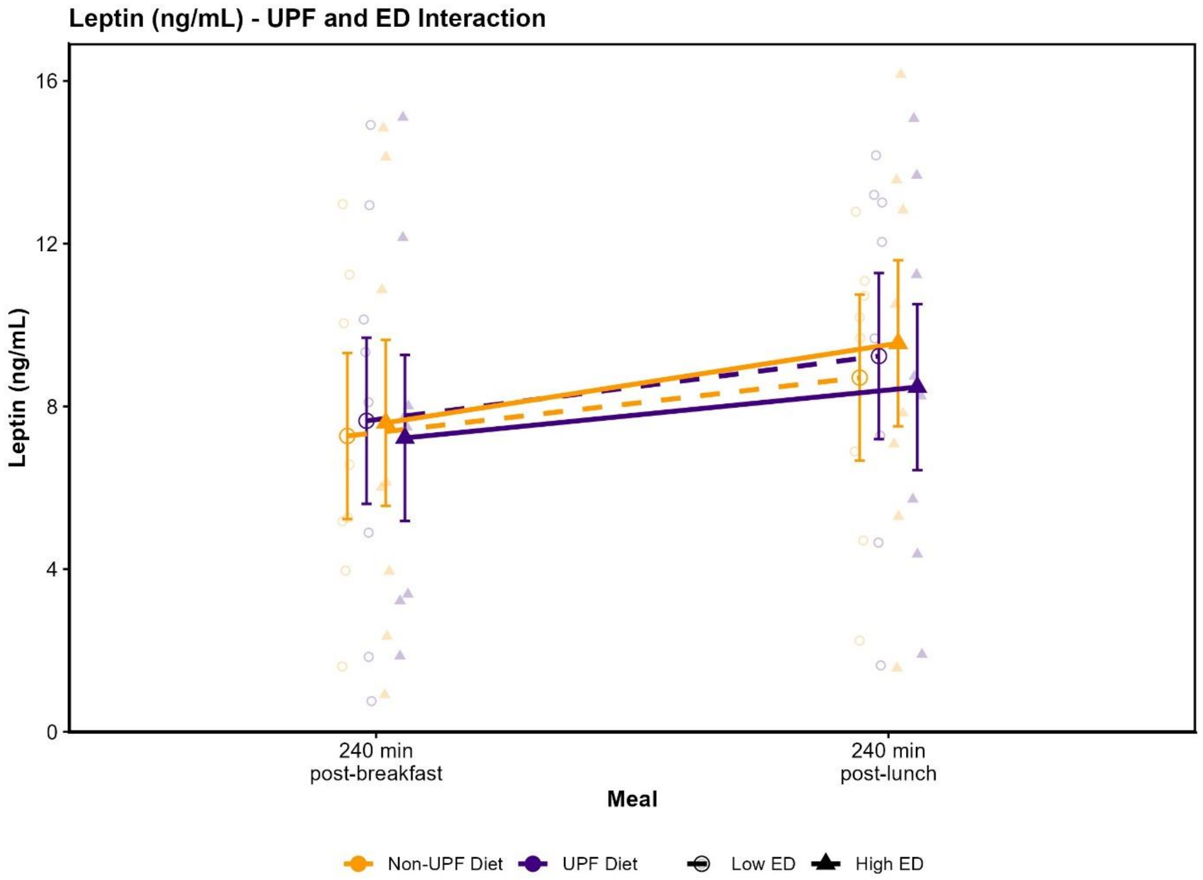

Supplementary Figure S14 Postprandial leptin (mg/dL) at 240 min after UPF and ED interaction meals (breakfast and lunch). The bold points represent estimated marginal means obtained from linear mixed models, and the error bars indicate 95% confidence intervals (95% CI). Semi-transparent points represent individual data.

Supplementary Table S1 Checklist of items to be included when reporting randomized crossover clinical trials (CONSORT).

Section/topic	No	CONSORT 2025 checklist item description	Reported on page no.
**Title and abstract**			
Title and structured abstract	1a	Identification as a randomised trial	2
	1b	Structured summary of the trial design, methods, results, and conclusions	2
**Open science**			
Trial registration	2	Name of trial registry, identifying number (with URL) and date of registration	4
Protocol and statistical analysis plan	3	Where the trial protocol and statistical analysis plan can be accessed	4
Data sharing	4	Where and how the individual de-identified participant data (including data dictionary), statistical code and any other materials can be accessed	N/A
Funding and conflicts of interest	5a	Sources of funding and other support (eg, supply of drugs), and role of funders in the design, conduct, analysis and reporting of the trial	18
	5b	Financial and other conflicts of interest of the manuscript authors	18
**Introduction**			
Background and rationale	6	Scientific background and rationale	3-4
Objectives	7	Specific objectives related to benefits and harms	4
**Methods**			
Patient and public involvement	8	Details of patient or public involvement in the design, conduct and reporting of the trial	4-11
Trial design	9	Description of trial design including type of trial (eg, parallel group, crossover), allocation ratio, and framework (eg, superiority, equivalence, non-inferiority, exploratory)	4-12
Changes to trial protocol	10	Important changes to the trial after it commenced including any outcomes or analyses that were not prespecified, with reason	N/A
Trial setting	11	Settings (eg, community, hospital) and locations (eg, countries, sites) where the trial was conducted	5
Eligibility criteria	12a	Eligibility criteria for participants	5
	12b	If applicable, eligibility criteria for sites and for individuals delivering the interventions (eg, surgeons, physiotherapists)	N/A

Intervention and comparator	13	Intervention and comparator with sufficient details to allow replication. If relevant, where additional materials describing the intervention and comparator (eg, intervention manual) can be accessed	5-12
Outcomes	14	Prespecified primary and secondary outcomes, including the specific measurement variable (eg, systolic blood pressure), analysis metric (eg, change from baseline, final value, time to event), method of aggregation (eg, median, proportion), and time point for each outcome	8-11
Harms	15	How harms were defined and assessed (eg, systematically, non-systematically)	N/A
Sample size	16a	How sample size was determined, including all assumptions supporting the sample size calculation	11
	16b	Explanation of any interim analyses and stopping guidelines	4-5
Randomisation:			
Sequence generation	17a	Who generated the random allocation sequence and the method used	6
	17b	Type of randomisation and details of any restriction (eg, stratification, blocking and block size)	6
Allocation concealment mechanism	18	Mechanism used to implement the random allocation sequence (eg, central computer/telephone; sequentially numbered, opaque, sealed containers), describing any steps to conceal the sequence until interventions were assigned	6
Implementation	19	Whether the personnel who enrolled and those who assigned participants to the interventions had access to the random allocation sequence	6
Blinding	20a	Who was blinded after assignment to interventions (eg, participants, care providers, outcome assessors, data analysts)	N/A
	20b	If blinded, how blinding was achieved and description of the similarity of interventions	N/A
Statistical methods	21a	Statistical methods used to compare groups for primary and secondary outcomes, including harms	12
	21b	Definition of who is included in each analysis (eg, all randomised participants), and in which group	13
	21c	How missing data were handled in the analysis	N/A
	21d	Methods for any additional analyses (eg, subgroup and sensitivity analyses), distinguishing prespecified from post hoc	N/A
**Results**			
Participant flow, including flow diagram	22a	For each group, the numbers of participants who were randomly assigned, received intended intervention, and were analysed for the primary outcome	13
	22b	For each group, losses and exclusions after randomisation, together with reasons	13
Recruitment	23a	Dates defining the periods of recruitment and follow-up for outcomes of benefits and harms	N/A

	23b	If relevant, why the trial ended or was stopped	N/A
Intervention and comparator delivery	24a	Intervention and comparator as they were actually administered (eg, where appropriate, who delivered the intervention/comparator, how participants adhered, whether they were delivered as intended (fidelity))	7-8
	24b	Concomitant care received during the trial for each group	N/A
Baseline data	25	A table showing baseline demographic and clinical characteristics for each group	13-15
Numbers analysed, outcomes and estimation	26	For each primary and secondary outcome, by group:<br>● the number of participants included in the analysis<br>● the number of participants with available data at the outcome time point<br>● result for each group, and the estimated effect size and its precision (such as 95% confidence interval)<br>● for binary outcomes, presentation of both absolute and relative effect size	
Harms	27	All harms or unintended events in each group	N/A
Ancillary analyses	28	Any other analyses performed, including subgroup and sensitivity analyses, distinguishing pre-specified from post hoc	N/A
**Discussion**			
Interpretation	29	Interpretation consistent with results, balancing benefits and harms, and considering other relevant evidence	15-18
Limitations	30	Trial limitations, addressing sources of potential bias, imprecision, generalisability, and if relevant, multiplicity of analyses	18
